# In utero and postnatal ivacaftor/lumacaftor therapy rescues multiorgan disease in *CFTR*-F508del ferrets

**DOI:** 10.1172/jci.insight.157229

**Published:** 2024-04-22

**Authors:** Idil Apak Evans, Xingshen Sun, Bo Liang, Amber R. Vegter, Lydia Guo, Thomas J. Lynch, Yan Zhang, Yulong Zhang, Yaling Yi, Yu Yang, Zehua Feng, Soo Yeun Park, Amanita Shonka, Hannah McCumber, Lisi Qi, Peipei Wu, Guangming Liu, Allison Lacina, Kai Wang, Katherine N. Gibson-Corley, David K. Meyerholz, Dominique H. Limoli, Bradley H. Rosen, Ziying Yan, Douglas J. Bartels, John F. Engelhardt

**Affiliations:** 1 Department of Anatomy and Cell Biology, and; 2 Department of Internal Medicine, University of Iowa Carver College of Medicine, Iowa City, Iowa, USA.; 3 Department of Biostatistics, University of Iowa College of Public Health, Iowa City, Iowa, USA.; 4 Department of Pathology, University of Iowa Carver College of Medicine, Iowa City, Iowa, USA.; 5 Department of Pathology, Microbiology and Immunology, Vanderbilt University Medical Center, Nashville, Tennessee, USA.; 6 Department of Microbiology and Immunology, University of Iowa Carver College of Medicine, Iowa City, Iowa, USA.

**Keywords:** Pulmonology, Therapeutics, Bacterial infections, Genetic diseases

## Abstract

Cystic fibrosis (CF) is caused by mutations in the CF transmembrane conductance regulator (*CFTR*) gene, with F508del being the most prevalent mutation. The combination of CFTR modulators (potentiator and correctors) has provided benefit to CF patients carrying the F508del mutation; however, the safety and effectiveness of in utero combination modulator therapy remains unclear. We created a F508del ferret model to test whether ivacaftor/lumacaftor (VX-770/VX-809) therapy can rescue in utero and postnatal pathologies associated with CF. Using primary intestinal organoids and air-liquid interface cultures of airway epithelia, we demonstrate that the F508del mutation in ferret CFTR results in a severe folding and trafficking defect, which can be partially restored by treatment with CFTR modulators. In utero treatment of pregnant jills with ivacaftor/lumacaftor prevented meconium ileus at birth in F508del kits and sustained postnatal treatment of CF offspring improved survival and partially protected from pancreatic insufficiency. Withdrawal of ivacaftor/lumacaftor treatment from juvenile CF ferrets reestablished pancreatic and lung diseases, with altered pulmonary mechanics. These findings suggest that in utero intervention with a combination of CFTR modulators may provide therapeutic benefits to individuals with F508del. This *CFTR*-F508del ferret model may be useful for testing therapies using clinically translatable endpoints.

## Abstract 2

A cystic fibrosis ferret model harboring the <em>CFTR</em>-F508del mutation was created to evaluate in utero and postnatal therapeutic efficacy of CFTR modulators.

## Graphical abstract

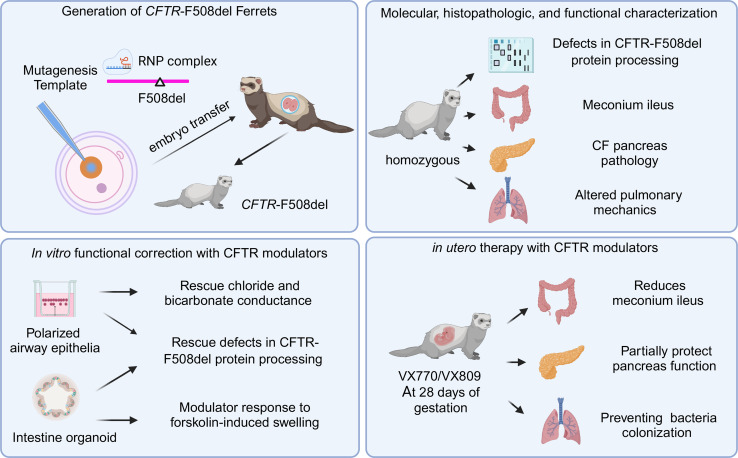

## Introduction

Cystic fibrosis (CF) is caused by mutations in the CF transmembrane conductance regulator (*CFTR*) gene, which encodes an anion channel that conducts chloride and bicarbonate across epithelia found in multiple organs (1, 2). *CFTR* mutations are classified into 6 categories according to their mechanism of dysfunction (1, 2). The class II mutation F508del is the most common *CFTR* mutation, with approximately 70% of CF patients having at least 1 copy (3, 4). The F508del mutation fails to generate a fully functional CFTR protein for 2 primary reasons: a gating defect that reduces anion flow through the channel pore and a folding defect that leads to premature degradation in the endoplasmic reticulum (ER) (5). Fortunately, small molecules called CFTR modulators (potentiators and correctors) have been developed to remedy both of these defects (1, 2).

CFTR correctors (lumacaftor [LUM], tezacaftor [TEZ], and elexacaftor [ELX]) enhance the folding and trafficking of the F508del CFTR mutant protein to the cell surface, while the CFTR potentiator ivacaftor (IVA) enhances gating of certain CFTR mutations (e.g., R117H, R334W, R347P, G551D, and F508del). Clinical studies have shown that double combination therapy, LUM/IVA or TEZ/IVA, provided therapeutic benefits for people with CF carrying 2 copies of the F508del mutation and F508del compound heterozygotes with 1 residual functional mutation, although improvements in lung function were relatively modest (6, 7). More recently, improved clinical results have been obtained with triple combinations of ELX/TEZ/IVA (ETI) that includes 2 correctors with complementary mechanisms of action (8).

CF disease initiates early in life, with common features including the poor hydration of mucous secretions that line epithelia of affected organs such as the lung, pancreas, intestine, gallbladder, liver, and male reproductive organs (2, 9). Lung disease is the most life-threatening aspect of CF and comorbidities such as CF-related diabetes and meconium ileus (MI) can negatively impact lung disease progression (10–12). Pancreatic disease leads to pancreatic insufficiency in most people with CF (13). MI, which is an obstruction of the intestine at birth, is a complication in approximately 20% of infants with CF and underscores that the consequences of CFTR dysfunction can initiate in utero (14). These aspects of the early and multiorgan nature of CF disease pathophysiology emphasize the need to initiate treatments at a very young age.

The youngest age of the most recent US Food and Drug Administration (FDA) approval for ETI use is 2 years, while the combination of LUM/IVA therapy is approved for use at 1 year (15). Although a recent phase III trial showed that LUM/IVA was generally safe and well tolerated in 1- to 2-year-old children homozygous for the F508del mutation, no CFTR modulators are currently approved for use during pregnancy (15). However, the number of pregnancies is on the rise in women with CF who are being treated with CFTR modulators (16). This has led to a number of case reports describing the birth of infants with CF who were exposed to CFTR modulators in utero and postnatally during nursing (17, 18). Therefore, there is a great need for an animal model capable of evaluating the efficacy of combination CFTR modulator therapy in utero.

CF animal models have aided the study of CF disease pathophysiology and the development of therapies, despite species-specific differences in organ pathologies (19, 20). Among these models, the *CFTR*^G551D/G551D^ ferret was the first to evaluate CFTR modulator therapy in utero and postnatally, demonstrating the safety and efficacy of IVA therapy to protect from MI, pancreatitis, and lung disease (21). Here, we describe the generation of the *CFTR*^F508del/F508del^ (hereinafter referred to as *CFTR*-F508del) ferret model (fCFTR-F508del) and evaluate the responsiveness of fCFTR-F508del to LUM/IVA in vivo and in vitro. This CFTR modulator–responsive *CFTR*-F508del ferret model may have utility in testing new CFTR therapies, and the long-term consequence of in utero exposure to CFTR combination modulator therapies.

## Results

### Generation of CFTR-F508del ferrets using zygote gene editing.

**Figure 1:**
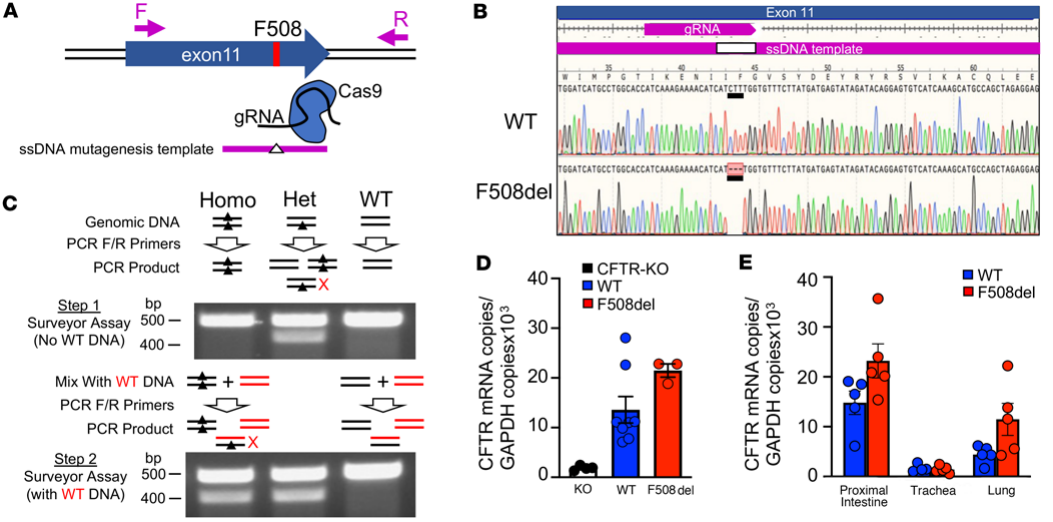
Generation of F508del-knockin ferret and characterization of *CFTR* mRNA expression. (**A**) Schematic of the targeting strategy at the ferret *CFTR* exon 11 locus using Cas9/gRNA complex and oligonucleotide mutagenesis template targeting deletion of F508. Forward (F) and reverse (R) primers used for amplification of the locus are marked by arrows. (**B**) Sequencing results of PCR-amplified genomic DNA, using F/R primers shown in **A**, from a heterozygous F508del founder. The sequence confirms deletion of the nucleotide triplet (CTT), which resulted in F508del. (**C**) Two-step genotyping protocol used to differentiate WT, *CFTR*-F508del heterozygous (Het), and *CFTR*-F508del homozygous (Homo) offspring. Step 1: The first Surveyor step distinguished a heterozygous genotype from homozygous and WT. Only the heterozygous PCR product is cleaved during the Surveyor assay due to the mismatch in bases (marked with X), giving rise to 2 bands on agarose gels. Step 2: To distinguish homozygous from WT genotypes, reference WT and test sample genomic DNA are mixed at a ratio of 0.5:1 (WT/Test) and PCR is then performed with F/R primers. These PCR products are then used in a second Surveyor assay. Only the homozygous PCR product is cleaved during the Surveyor assay due to the mismatch in bases (marked with X), giving rise to 2 bands on agarose gels. (**D**) qPCR analysis for *CFTR* mRNA on ferret intestinal organoids generated from *CFTR*-KO (*n* = 4 donors), *CFTR*-WT (*n* = 8 donors), and *CFTR*-F508del homozygous (*n* = 3 donors) ferrets. Data represent mean ± range. (**E**) qPCR analysis for *CFTR* mRNA from proximal intestine (*n* = 5 donors), trachea (*n* = 4 donors for WT and *n* = 5 donors for F508del), and lung (*n* = 5 donors) from *CFTR*-WT and *CFTR*-F508del newborn ferrets. Data represent mean ± SEM.

*CFTR*-F508del ferrets were generated by injection of zygotes with a Cas9/sgRNA ribonuclear protein complex and a single-stranded DNA homology template, both targeting exon 11 of the ferret *CFTR* gene (Figure 1A). The deletion of the F508 codon was confirmed by Sanger sequencing in founder offspring DNA (Figure 1B) and a PCR-based T7 endonuclease Surveyor assay was developed to distinguish *CFTR*^+/+^, *CFTR*^+/F508del^, and *CFTR*^F508del/F508del^ animals (Figure 1C). To confirm that expression from the F508del allele was unaffected by the mutation, we performed a reverse transcription–quantitative PCR (RT-qPCR) on intestinal organoid mRNA derived from newborn *CFTR*^+/+^ (WT), *CFTR*^–/–^ (*CFTR*-KO), and *CFTR*-F508del animals. *CFTR* mRNA levels were indistinguishable between WT and *CFTR*-F508del organoids and above that of *CFTR*-KO negative controls (Figure 1D). Similarly, *CFTR* mRNA in *CFTR*-F508del newborn intestine, trachea, and lung tissues was equal to or greater than that of WT animals (Figure 1E). Not surprisingly, *CFTR* mRNA was the lowest in the trachea since CFTR-rich submucosal glands develop postnatally in the ferret.

### CFTR modulators partially rescue ferret CFTR-F508del protein-processing defects in intestinal organoids and polarized airway epithelia.

**Figure 2:**
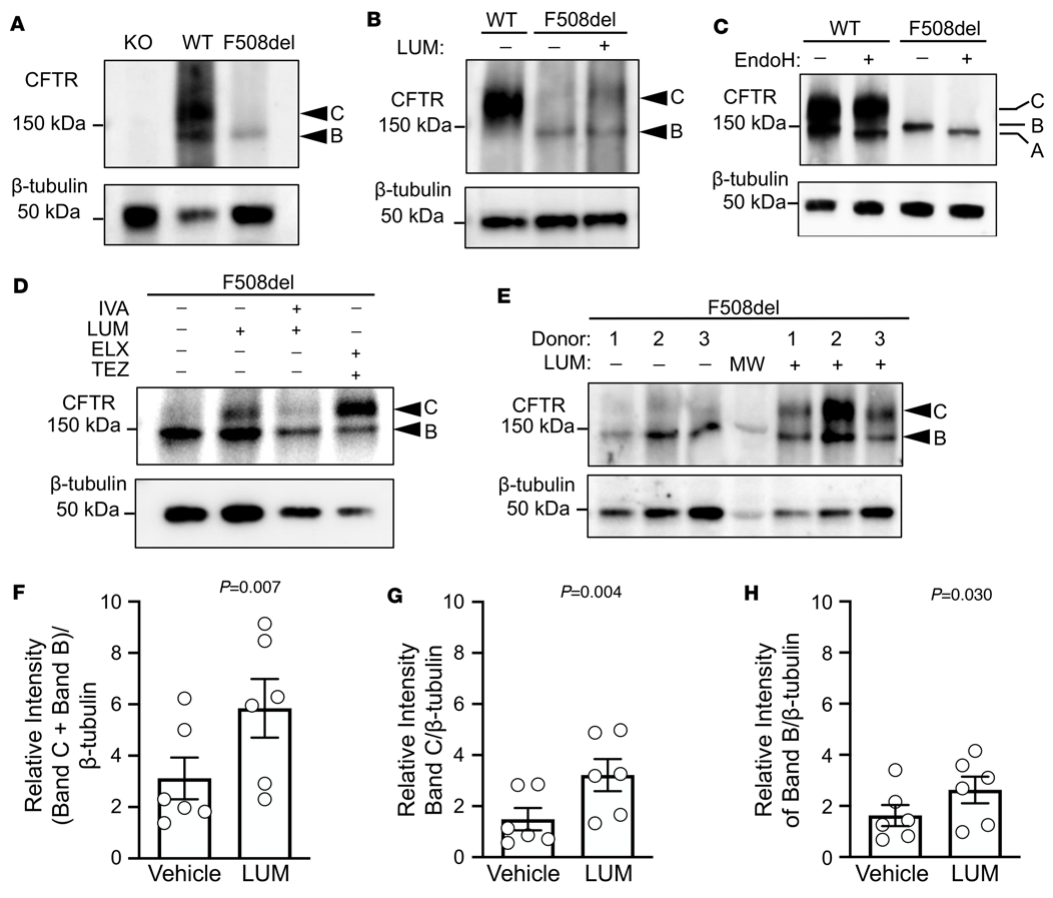
Ferret F508del CFTR processing in intestinal organoids and differentiated air-liquid interface (ALI) cultures of airway epithelia. (**A**) Western blots of CFTR and β-tubulin protein expression from ferret intestinal organoids of WT, *CFTR-*KO (KO), and *CFTR*-F508del homozygous genotypes. Band C indicates the mature (complex glycosylated) form of CFTR and Band B indicates the immature (core glycosylated) form of CFTR. (**B**) Western blots of CFTR and β-tubulin protein expression from differentiated airway epithelia grown at an ALI generated from WT and F508del (homozygous) ferret tracheal basal cells. F508del cells were treated overnight with either vehicle (DMSO) or LUM (3 μM). (**C**) Western blot of WT and F508del ferret intestinal organoid lysates incubated with or without endoglycosidase H (EndoH) to cleave N-linked glycosylated residues added in the ER. Susceptibility to EndoH cleavage indicates ER-resident CFTR (Band B) and produces the slightly smaller Band A form of CFTR. The fully mature form of CFTR (Band C) is not sensitive to EndoH. The majority of F508del CFTR protein is thus resident in the ER. (**D**) F508del ferret organoids were incubated at 37°C for 16 hours in the presence of vehicle (DMSO), LUM (CFTR corrector), IVA (CFTR potentiator) and LUM, or ELX and TEZ (CFTR correctors) at 3 μM final concentration. (**E**) F508del homozygous differentiated airway cultures grown at an ALI were incubated with LUM (3 μM, 16 hours at 37°C) and then evaluated by Western blotting. Three independent donor animals were used per condition. (**F**–**H**) Quantification of the relative intensity of CFTR forms normalized to β-tubulin in differentiated airway cultures treated with vehicle (DMSO) or LUM: (**F**) (Band C + Band B)/β-tubulin, (**G**) Band C/β-tubulin, and (**H**) Band B/β-tubulin. Data represent mean ± SEM. Statistical significance evaluated by paired 2-tailed Student’s *t* test (*n* = 6 Transwells, reflecting 2 replicates from each of 3 independent donors). *P* values are given in each panel.

Earlier studies elucidating the processing and electrophoretic mobility of CFTR protein have shown 3 different forms distinguished by molecular weight: 127 kDa (no glycosylation, Band A), 131 kDa (core-glycosylated immature protein, Band B), and 170 kDa (complex glycosylated mature protein, Band C) (22). The F508del mutation leads to a misfolded CFTR that alters the stability of the protein, leading to ER-association degradation (ERAD) (23, 24). We evaluated the processing of ferret CFTR-F508del in intestinal organoids and polarized airway epithelia grown in air-liquid interface (ALI) cultures. As expected, the CFTR Band C predominated in WT organoids and *CFTR-KO* cultures lacked immunoreactivity (Figure 2A). CFTR-F508del intestinal organoids (Figure 2A) and airway ALI cultures (Figure 2B) predominantly expressed Band B. Overnight treatment with LUM increased the expression of Band C in CFTR-F508del intestinal organoids (Figure 2B).

To confirm Band B was the core-glycosylated form, we used endoglycosidase H (EndoH), which specifically cleaves the N-glycan residues found only in Band B. As expected, Band C migration was not affected by EndoH treatment in WT organoids, confirming that this band represents the fully glycosylated mature form of CFTR (Figure 2C). By contrast, Band B in both WT and *CFTR*-F508del organoids was cleaved by EndoH, giving rise to a slightly smaller nonglycosylated form of CFTR (Band A). These results confirm that the CFTR-F508del protein resides primarily in the ER as the Band B core-glycosylated form.

To test the effectiveness of correctors and potentiators, we treated WT and CFTR-F508del intestinal organoids and airway ALI cultures for 16 hours with IVA, LUM, ELX, and/or TEZ. Exposure of CFTR-F508del intestinal organoids to LUM or ELX/TEZ enhanced processing to Band C, with the combination being more effective than LUM alone (Figure 2D). Notably, ferret CFTR-F508del organoids exposed for 16 hours to the combination of LUM/IVA led to impaired accumulation of Band C, as compared with LUM alone (Figure 2D), similar to previous results in human CFTR-F508del epithelial cells (25). LUM treatment of airway ALI cultures also significantly enhanced ferret CFTR-F508del processing to Band C (Figure 2, E–H). These results indicated that the ferret CFTR-F508del protein has a severe folding and trafficking defect, which can be rescued by CFTR correctors.

### Ferret CFTR-F508del intestinal organoids are functionally responsive to LUM/IVA in forskolin-induced swelling assays.

**Figure 3:**
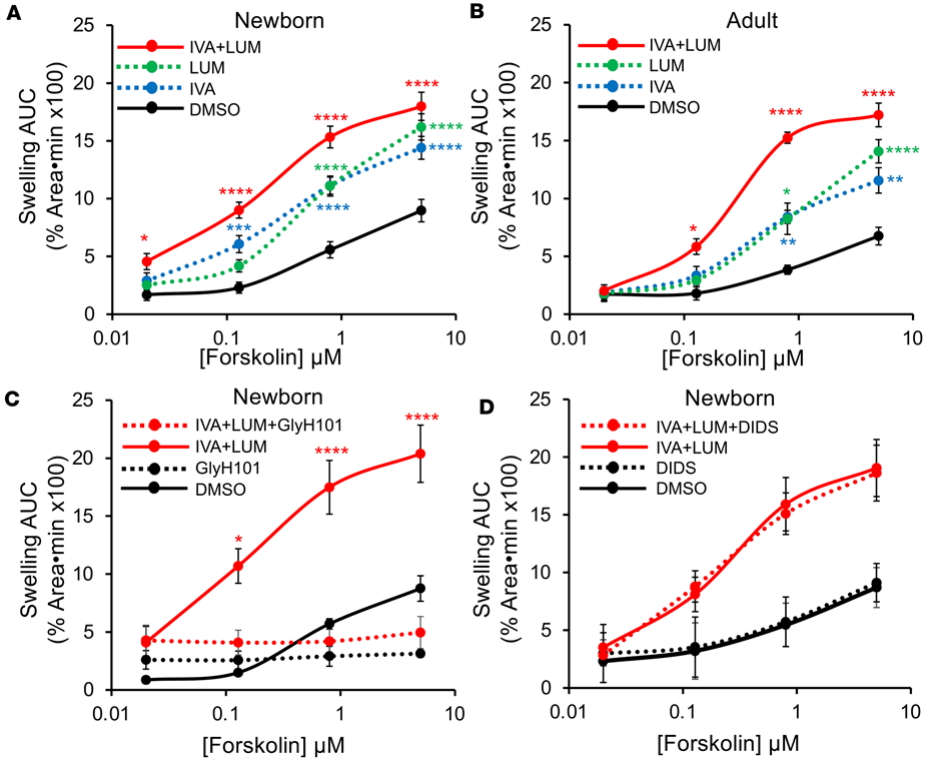
Combination LUM/IVA treatment enhances forskolin-induced swelling responses of ferret *CFTR*-F508del intestinal organoids. (**A**) Forskolin-induced swelling (FIS) assays on homozygous *CFTR*-F508del newborn ferret intestinal organoids in the presence of DMSO, IVA (3 μM), LUM (3 μM), or LUM/IVA (3 μM each). Each point represents the average area under the curve (AUC) for *n* = 17 cultures, reflecting 1–3 replicates from 14 donors. (**B**) FIS assays on homozygous *CFTR*-F508del adult ferret intestinal organoids in the presence of DMSO, IVA (3 μM), LUM (3 μM), or LUM/IVA (3 μM each) (*n* = 5 cultures, reflecting 1–2 replicates from 3 donors). (**C** and **D**) FIS assay on homozygous *CFTR*-F508del newborn ferret intestinal organoids in the presence and absence of (**C**) GlyH101 (*n* = 4 cultures, reflecting 1–2 replicates from 3 donors) and (**D**) DIDS (*n* = 5 cultures, reflecting 1–2 replicates from 4 donors). All data represent mean ± SEM. Significant differences were determined by 2-way ANOVA with Bonferroni’s post hoc test. *P < 0.05*;* **P < 0.01; ***P < 0.001; ****P < 0.0001 for the following comparisons: (**A** and **B**) IVA, LUM, and LUM/IVA treatment groups versus DMSO controls; (**C**) LUM/IVA versus LUM/IVA/GlyH101 or DMSO versus GlyH101 (no significant differences); and (**D**) LUM/IVA versus LUM/IVA/DIDS or DMSO versus DIDS, which demonstrated no significant differences.

To verify functional rescue of CFTR-F508del by CFTR modulators, we performed forskolin-induced swelling (FIS) assays on intestinal organoids. Newborn (Figure 3A) and adult (Figure 3B) ferret CFTR-F508del organoids retained a small amount of residual CFTR function beginning at approximately 0.5 μM forskolin in the absence of CFTR modulators, similar to human *CFTR*-F508del rectal organoids (26). The combination of LUM/IVA produced the largest increases in FIS of CFTR-F508del organoids, as compared with the vehicle-treated (DMSO) controls (Figure 3, A and B), while IVA or LUM alone more moderately increased swelling. Compared with previously reported studies (21), LUM/IVA treatment of CFTR-F508del organoids had a swelling response that was 75% and 73% of that of WT ferret organoids at 5 μM and 0.8 μM forskolin, respectively. To evaluate whether the swelling responses observed were solely due to CFTR function, we performed studies with 2 anion channel antagonists: (a) GlyH101 to block CFTR and (b) 4,4′-diisothiocyanatostilbene-2,2′-disulfonic acid (DIDS) to block certain other non-CFTR anion channels. The FIS responses to LUM/IVA were abolished by the addition of GlyH101 (Figure 3C), whereas the addition of DIDS did not alter FIS responses to LUM/IVA (Figure 3D). Collectively, these results demonstrate that CFTR-F508del is responsive to LUM/IVA.

### CFTR modulators partially rescue chloride and bicarbonate conductance in polarized airway epithelia of CFTR-F508del ferrets.

**Figure 4:**
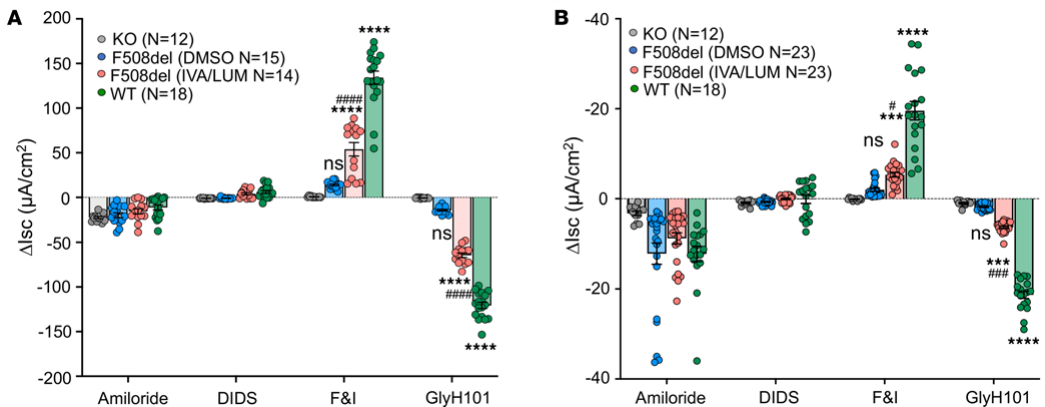
Short circuit (Isc) measurements of ferret *CFTR*-F508del differentiated airway epithelia grown at an air-liquid interface. Differentiated airway epithelia were generated using tracheal basal cell cultures derived from *CFTR*-KO (KO), homozygous *CFTR*-F508del (F508del), and WT ferrets and polarized at an air-liquid interface (ALI) for 3 weeks. Ussing chamber measurements of Isc were performed under (**A**) bicarbonate-free symmetric-chloride buffer to assess chloride currents and (**B**) chloride-free symmetric-bicarbonate buffer to assess bicarbonate current. Graphs show the change in Isc (ΔIsc) following the sequential addition of amiloride, 4,4′-diisothiocyanatostilbene-2,2′-disulfonic acid (DIDS), forskolin and 3-isobutyl-1-methylxanthine (F&I), and GlyH101 (CFTR inhibitor). Data represent the mean ± SEM for the indicated number (*n*) of Transwells measured for each condition (6–8 Transwells assayed for each of 2–3 donors per genotype). Significant differences were determined by 1-way ANOVA with Bonferroni’s multiple-comparison test. ***P < 0.001, ****P < 0.0001 for comparison of F508del and WT to KO; ^#^*P* < 0.05, ^###^*P* < 0.001, ^####^*P* < 0.0001 for comparison of F508del LUM/IVA treatment to F508del vehicle (DMSO) treatment.

To evaluate the impact of CFTR modulators on the function of CFTR-F508del in airway epithelia, we generated differentiated ALI cultures from primary tracheal epithelial cells isolated from *CFTR*-KO, *CFTR*-F508del, and WT ferrets. CFTR-dependent chloride and bicarbonate short circuit currents (Isc) were assessed separately using symmetrical chloride (bicarbonate-free) and bicarbonate (chloride-free) buffers, respectively. *CFTR*-F508del ferret ALI cultures treated with vehicle (DMSO) alone produced Cl^–^ and HCO_3_^–^ currents in response to forskolin plus 3-isobutyl-1-methylxanthine (IBMX) that were approximately 10% of WT ALI cultures and the currents were inhibited by the CFTR channel blocker GlyH101, whereas *CFTR*-KO cultures lacked these currents (Figure 4). This level of residual Cl^–^ current is approximately double that of human F508del-homozygous ALI cultures (27) and consistent with slightly increased processing of recombinant ferret CFTR*-*F508del protein in cell lines (28). Treatment of ferret CFTR-F508del ALI cultures with LUM/IVA significantly increased forskolin/IBMX– and GlyH101-responsive changes to Cl^–^ currents, achieving 40% and 53% of those observed in WT ALI cultures, respectively. The impact of CFTR modulators on HCO_3_^–^ currents was less than that for Cl^–^ currents, with LUM/IVA treatment achieving currents that were 27% and 30% that of WT cultures in response to forskolin/IBMX or GlyH101, respectively.

### In utero and postnatal LUM/IVA treatment reduces MI and improves the survival of CFTR-F508del ferrets.

**Figure 5:**
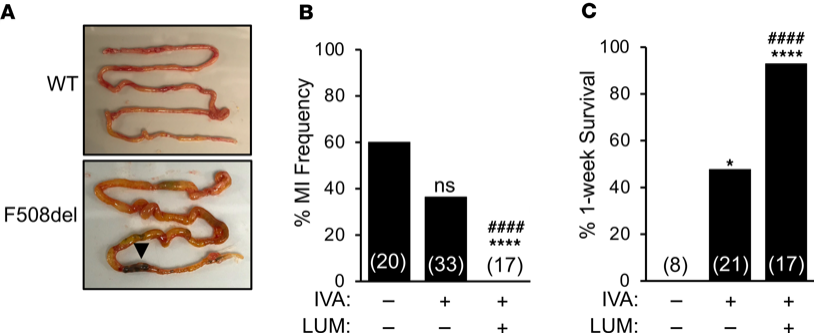
In utero and postnatal LUM/IVA treatment reduces meconium ileus and improves the survival of *CFTR*-F508del newborn ferrets. (**A**) Representative images of the intestine from newborn kits with the indicated homozygous genotypes. Arrow marks the intestinal obstruction in the *CFTR*-F508del kit with meconium ileus (MI). (**B**) Percentage of newborn homozygous *CFTR*-F508del kits with MI at birth with the indicated in utero treatment conditions. Pregnant jills harboring *CFTR*-F508del kits were untreated or treated with IVA or LUM/IVA beginning on embryonic day 28 (E28). Numbers in parentheses represent the number of births for each treatment condition evaluated. (**C**) One-week survival rates of homozygous *CFTR*-F508del kits that passed stool at birth for the indicated in utero and postnatal treatment conditions. In **B** and **C**, Fisher’s exact test was used to compare the untreated group to each of the treatment groups (NS, *P* = 0.154, *P = 0.0265, ****P < 0.0001) or the IVA-treated to the LUM/IVA–treated groups (^####^*P* < 0.0001).

MI is one of the earliest pathologic manifestations of CF, affecting approximately 20% of CF infants (14). *CFTR*-KO ferrets have an MI incidence of approximately 80% (29), but in utero IVA treatment of *CFTR*^G551D/G551D^ ferrets reduces the frequency of MI to approximately 4% (21). To further test the therapeutic potential of IVA alone and LUM/IVA combined, we treated *CFTR*-F508del ferrets on embryonic day 28 of the 42-day gestation period. In the absence of CFTR modulator therapy, 60% of *CFTR*-F508del ferrets developed MI (Figure 5, A and B). In utero treatment with IVA alone nonsignificantly reduced the MI frequency to 38.5%; however, the LUM/IVA combination provided full protection from MI, with 100% passing meconium at birth (*P* < 0.0001) (Figure 5B). Continued postnatal treatment with IVA or LUM/IVA significantly increased 1-week survival rates of non-MI *CFTR*-F508del kits to 48% (*P* = 0.0265) and 93% (*P* < 0.0001), respectively, while there was 0% survival in the untreated group (Figure 5C). Overall, these studies show that in utero and postnatal LUM/IVA treatment of *CFTR*-F508del ferrets has the greatest effect on alleviating MI and improving early survival rates. The partial therapeutic benefit in 1-week survival of CF kits on IVA alone suggests that a low level of CFTR-F508del at the plasma membrane can be functionally potentiated.

### In utero and postnatal treatment with LUM/IVA partially protects the CFTR-F508del ferret pancreas from exocrine destruction.

*CFTR*-KO ferrets are born with relatively minor pancreatic pathology at birth; however, rapid destruction of the exocrine pancreas and islet remodeling ensues over the first 2 months of life (30, 31). Similar to newborn *CFTR*-KO kits, the most visually evident pathological findings in *CFTR*-F508del kits were mild to moderate exocrine acinar duct dilation with homogeneous eosinophilic secretory material and edematous pancreatic interstitial tissue (Supplemental Figure 1; supplemental material available online with this article; https://doi.org/10.1172/jci.insight.157229DS1). Islet morphology was relatively intact in *CFTR*-F508del kits and similar to CFTR-KO kits (30).

**Figure 6:**
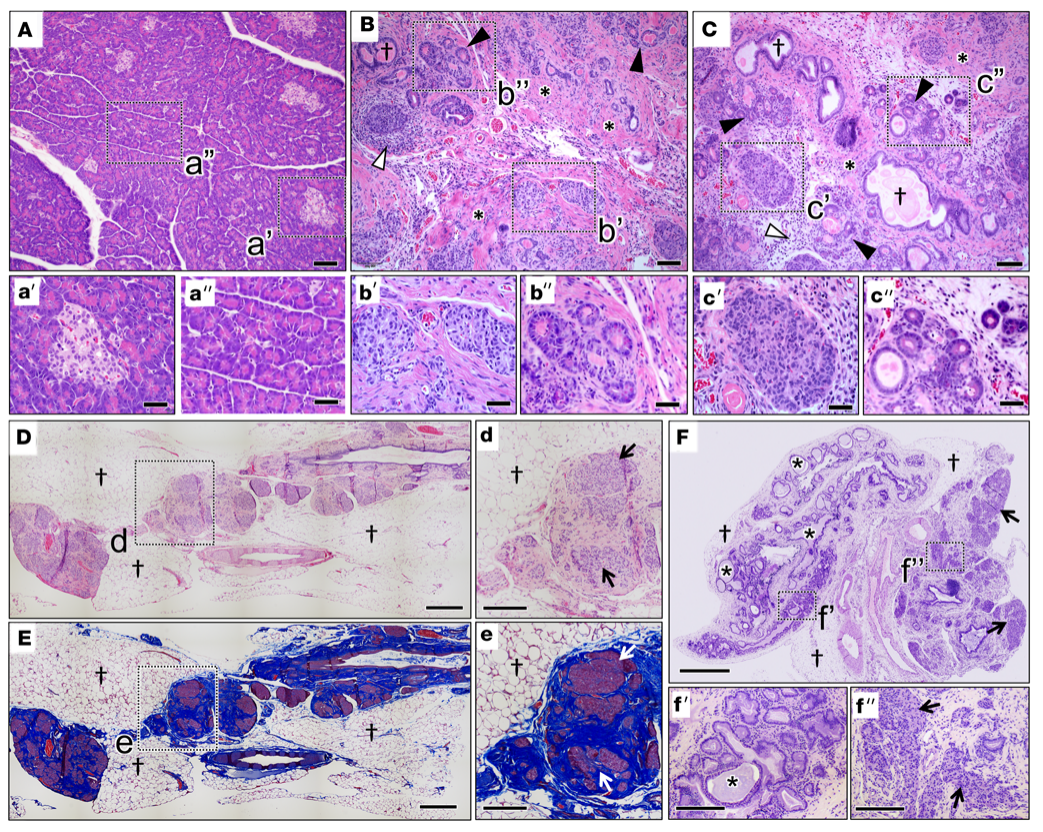
Pancreatic pathology of juvenile and adult WT and *CFTR*-F508del ferrets. (**A**–**C**) H&E-stained sections of the pancreas from 2-month-old (**A**) WT and (**B** and **C**) 2 homozygous *CFTR*-F508del ferrets. These *CFTR*-F508del ferrets received only IVA in utero and postnatally until sacrifice at 2 months of age. Note the marked pancreatic fibrosis (*), acinar loss, cystic dilations (closed arrows), ductal dilatation (†), and pockets of inflammation (open arrows). Islet morphology was relatively intact, as shown in the boxed regions of the main panels and enlarged insets (a’–c’). Residual acinar cells with cystic dilatations are shown in the boxed regions of the main panels and enlarged insets (a”–c”). (**D**–**F**) Sections of the pancreas from 2 different adult *CFTR*-F508del ferrets that were reared on only IVA, later removed, and euthanized at more than 1 year of age. Insets show enlarged boxed regions in the main panels (d, e, f’, and f’’). Note the marked adipose replacement (†), ductal dilatation (*), and aggregated islets in pockets of fibrosis (arrows). (**E** and e) Masson’s trichrome staining for fibrosis (blue). Scale bars: 100 μm (**A**–**C**), 50 μm (a–c), 1 mm (**D**), and 250 μm (d).

In utero and postnatal administration of IVA alone failed to protect the *CFTR*-F508del ferret pancreas from pathologies (Figure 6). *CFTR*-F508del ferrets maintained on IVA for the first 2 months of life presented extensive pancreatic fibrosis, cystic duct dilation, acinar loss, and pockets of inflammation (Figure 6, B and C), similar to that observed in CFTR-KO ferrets (30, 31). Some small pockets of residual acinar cells remained and islets were more intact (Figure 6, A–C) than that of age-matched *CFTR*-KO ferrets (30, 31). Adult *CFTR*-F508del ferret pancreata were also evaluated in ferrets reared on IVA during the early neonatal to juvenile period and then removed from the drug (Figure 6, D–F). Pancreata from these adult *CFTR*-F508del ferrets (Figure 6, D–F) had a greater extent of fat accumulation, ductal hyperplasia with mucus accumulation in the lumen, and islet aggregation in the pancreas as compared with juvenile *CFTR*-F508del ferrets (Figure 6, B and C). Histopathology in the gallbladder of these adult *CFTR*-F508del ferrets was also noted, including mucosal thickening and cystic structures containing periodic acid–Schiff–stained (PAS-stained) mucus (Supplemental Figure 2).

**Figure 7:**
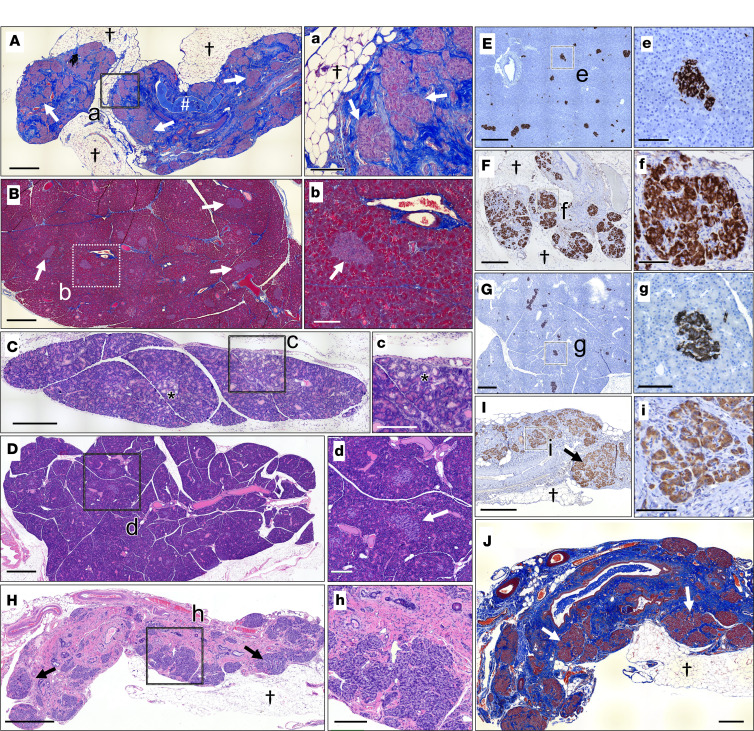
In utero and postnatal LUM/IVA treatment of *CFTR*-F508del ferrets partially protects from pancreatitis. (**A**) Masson’s trichrome–stained section of the pancreas from a *CFTR*-F508del ferret treated in utero and postnatally with LUM/IVA until 45 days of age and sacrificed at 101 days of age. There is marked pancreatic fibrosis (blue staining), adipose replacement (†), duct plugging (#), and islet aggregation (arrows). (**B** and **C**) Masson’s trichrome– (**B**) and H&E-stained (**C**) sections of the pancreas from a *CFTR*-F508del ferret treated in utero and postnatally with LUM/IVA until sacrificed at 51 days of age. The H&E-stained section highlights a localized area with cystic dilatation of acini and inflammatory infiltrates (*) in the animal continuously treated with LUM/IVA. When quantified, this type of pathology was observed in 4.1% of the area of the entire pancreas. Islets are marked by arrows and have normal architecture. (**D**) H&E-stained section of the pancreas from the 223-day-old *CFTR*-F508del ferret continuously treated with LUM/IVA. This ferret had normal fecal elastase levels at 140 days (4,682 μg EL-1/g feces) and showed no areas of acinar loss in the head, body, and tail of the pancreas and normal islet architecture (arrow). (**E**–**G**) Islet organization shown by histochemical staining of pancreatic sections for insulin (brown) in adult (**E**) WT (500 days old), (**F**) *CFTR*-F508del (505 days old) removed from LUM/IVA at 117 days, and (**G**) *CFTR*-F508del (223 days old) reared on LUM/IVA until euthanized. (**H**–**J**) Pancreatic sections stained with (**H**) H&E, (**I**) for insulin, and (**J**) for insulin with Masson’s trichrome from a CFTR-F508del ferret treated in utero and postnatally with LUM/IVA until 45 days of age and euthanized at 296 days of age. This ferret had undetectable fecal elastase levels before the necropsy. Aggregated islets are marked by an arrow and † marks fat. (a–i) Higher-power magnification images of the boxed regions in the main panels (**A**–**I**). Scale bars: 1 mm (**A**, **B**, **D**, **H**, and **J**), 500 μm (**C**, **E**–**G**, and **I**), 250 μm (a–d and h), and 100 μm (e–g and i).

In contrast with IVA monotherapy, the combination of LUM/IVA provided nearly full protection from CF pancreatic pathologies in 40% of *CFTR*-F508del ferrets. Two of the 5 *CFTR*-F508del ferrets maintained on LUM/IVA demonstrated pancreatic histology near (Figure 7, B and C) or within normal limits (Figure 7D) at 51 and 223 days of age, respectively. Pancreatic histopathology in animals removed from LUM/IVA at approximately 45 days of age and sacrificed at terminal disease demonstrated complete acinar loss, extensive fibrosis, and fat accumulation (Figure 7, A, H, and J), similar to adult *CFTR*-KO ferrets (31). Furthermore, the 2 pancreatic sufficient *CFTR*-F508del ferrets maintained on LUM/IVA retained islets with normal architecture similar to WT animals (Figure 7, E and G), while insulin-expressing islets aggregated in fibrotic regions of the pancreas in *CFTR*-F508del ferrets removed from LUM/IVA therapy (Figure 7, F and I).

### Spontaneous bacterial colonization of the lung in CFTR-F508del ferrets after the termination of LUM/IVA therapy.

**Figure 8:**
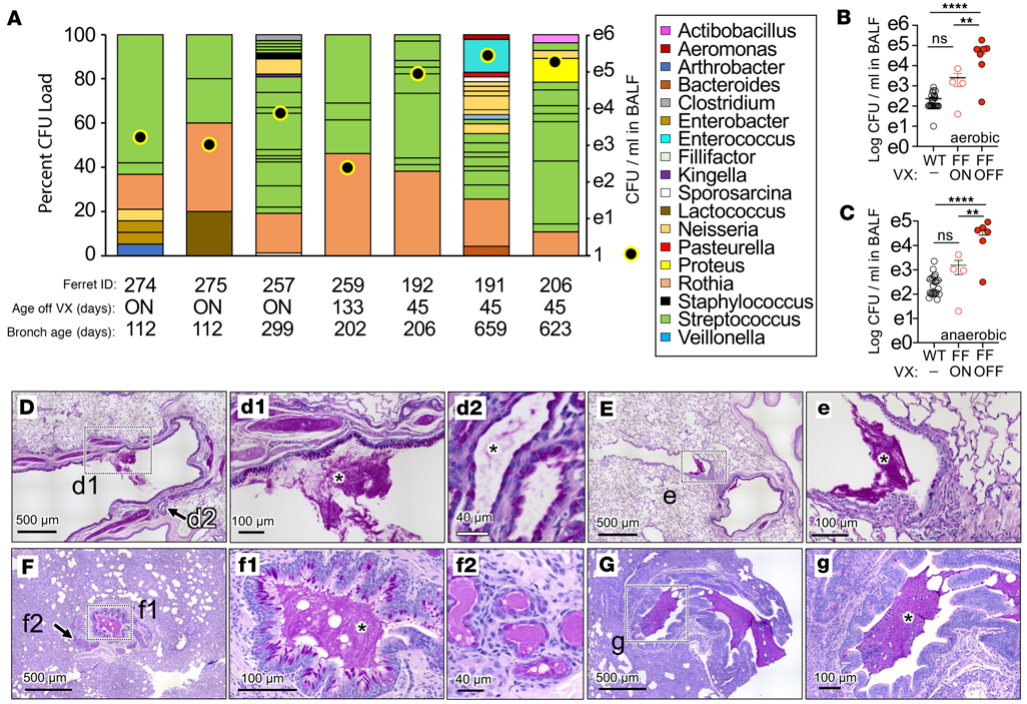
Bacterial colonization of the lung in WT and *CFTR*-F508del ferrets. (**A**) *CFTR*-F508del (FF) ferrets were treated in utero and postnatally with LUM/IVA (VX) until the indicated ages and bronchoalveolar lavage fluid (BALF) collected at the age indicated to assess bacterial load in the BALF and bacterial genus. The percentage of each bacterial genus is given on the left axis, with the color code given in the legend. The bacterial load is given on the right axis as colony-forming units (CFU) per mL. “ON” in **A** indicates that these 3 ferrets were treated with LUM/IVA for the entire time. (**B** and **C**) Longitudinal bronchoscopies of *CFTR*-F508del and WT ferrets assessing aerobic (**B**) and anaerobic (**C**) bacterial CFU in the BALF of independent ferrets shown in **A**. Significant differences were determined by 1-way ANOVA with Tukey’s multiple-comparison test. **P < 0.01, ****P < 0.0001 for comparison between WT and ON/OFF LUM/IVA *CFTR*-F508del ferret groups. NS, *P* > 0.05. Each animal underwent 1–4 bronchoscopies. (**D**–**G**) PAS-stained sections of inflated lungs from a *CFTR*-F508del ferret (**D** and **E**) reared on only IVA from E28 to 117 days of age, and then removed and sacrificed at 505 days of age and (**F** and **G**) reared on both IVA and LUM from E28 to 133 days of age, and then removed and sacrificed at 136 days of age due to acute health concerns. Mucus accumulation (asterisk) in the airways (d1, e, f1, and g) and submucosal glands (d2 and f2) are marked by asterisks and arrows. Boxed regions and arrow in the main panel point to areas of enlarged insets. Scale bars: 500 μm (**D**–**G**), 100 μm (d1, e, f1, and g), and 40 μm (d2 and f2).

To evaluate the effectiveness of LUM/IVA therapy in preventing the development of lung disease, we assessed the bacterial flora in the bronchioalveolar lavage fluid (BALF) of *CFTR*-F508del ferrets. Animals that were maintained on LUM/IVA had average CFU counts that were not significantly different from those of WT animals (Figure 8, A–C). By contrast, animals that had their LUM/IVA treatment terminated exhibited significantly higher CFU counts (both aerobic and anaerobic) in their BALF compared with both WT animals (*P* < 0.0001) and *CFTR*-F508del ferrets maintained on LVA/LUM (*P* < 0.01) (see Figure 8, A–C). With 15 genera identified, *Streptococcus* was the predominant bacteria found in all CF animals (either modulator-maintained or modulator-terminated).

After the termination of LUM/IVA treatment, the health of *CFTR*-F508del ferrets declined at various times. One animal (Figure 8, F and G) acutely declined 3 days following modulator withdrawal at 133 days of age. Additionally, 4 *CFTR*-F508del ferrets became acutely ill at approximately 2 months following the withdrawal of CFTR modulators at 45 days of age. These 4 acutely ill *CFTR*-F508del animals exhibited blood in the airways of one or more lobes, with partial airway obstruction due to hemorrhage, fibrin, scattered polymorphonuclear leukocytes (PMNs), and mucus (Supplemental Figure 3). Epithelial injury (erosion, ulceration, and necrosis) was observed in the lower left lobe of 3 animals. Mucus accumulation in the airways and the submucosal gland was observed in *CFTR*-F508del ferrets removed from modulators, which ranged from mild (Figure 8, D and E) to moderate (Figure 8, F and G) and often localized to specific lobes of the lung.

### CFTR-F508del ferrets have altered pulmonary mechanics.

**Figure 9:**
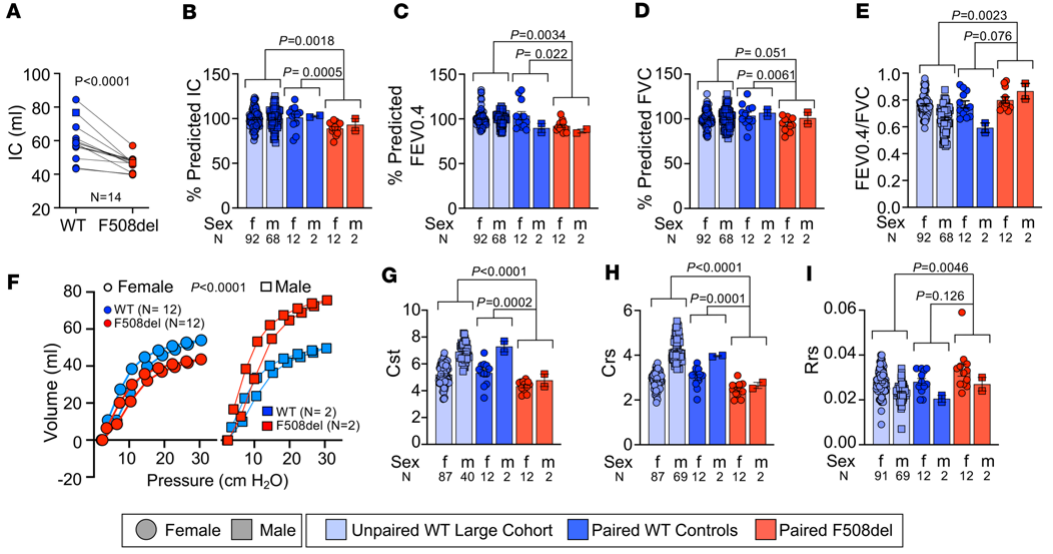
*CFTR*-F508del ferrets have altered respiratory mechanics indicative of obstructive and restrictive lung disease. FlexiVent pulmonary function testing was performed on 3 groups of ferrets: (i) *CFTR*-F508del ferrets (*n* = 14 measurements in 6 animals), (ii) WT ferrets that were paired controls in terms of age and sex (*n* = 14 measurements in 13 animals), and (iii) a larger cohort of WT ferrets (*n* = 160 measurements from a total of 68 animals). The average age of the paired cohort was 334 ± 49 days for *CFTR*-F508del and 388 ± 74 days for WT. The average age of the larger WT cohort was 328 ± 16 days. Data are shown as mean ± SEM and broken down by sex, with the number of measurements (*n*) used for each parameter. A mixed-effects model, which accounted for repeat measures, was used to compare genotypes and calculate *P* values. (**A**) Inspiratory capacity (IC) from age- and sex-matched pairs of WT and *CFTR*-F508del ferrets (*P* < 0.0001). (**B**) Percentage predicted IC (matched pairs, *P* = 0.0005; larger cohort, *P* = 0.0018). (**C**) Percentage predicted forced expiratory volume in 0.4 seconds (FEV_0.4_) (matched pairs, *P* = 0.022; larger cohort, *P* = 0.0034). (**D**) Percentage predicted forced vital capacity (FVC) (matched pairs, *P* = 0.0061; larger cohort, *P* = 0.051). (**E**) FEV_0.4_/FVC ratio (matched pairs, *P* = 0.076; larger cohort, *P* = 0.0023). (**F**) Pressure-volume loops (PV-loops) between pairs of WT and *CFTR*-F508del ferrets (*P* < 0.0001 for grouped analysis of both sexes). (**G**) Quasistatic compliance (Cst) (matched pairs, *P* = 0.0002; larger cohort, *P* < 0.0001). (**H**) Dynamic compliance (Crs) (matched pairs, *P* = 0.0001; larger cohort, *P* < 0.0001). (**I**) Resistance of the respiratory system (Rrs) (matched pairs, *P* = 0.126; larger cohort, *P* = 0.0046). The age and CFTR modulator parameters of F508del animals in the paired cohort can be found in the Supporting Data Values file for **A**.

Progressive airflow obstruction by mucus plugging, inflammation, and scarring (fibrosis) alters pulmonary mechanics in people with CF. To assess this, we performed pulmonary function tests (PFTs) on *CFTR*-F508del ferrets. Results showed that inspiratory capacity (IC) was significantly (*P* < 0.0001) reduced in *CFTR*-F508del ferrets when compared with their age- and sex-matched WT cohort (Figure 9A). Using a larger WT control cohort for calculating percentage predicted values, percentage predicted IC was also reduced in *CFTR*-F508del ferrets compared with both the larger (*P* *=* 0.0018) and paired (*P* *=* 0.0005) WT control cohorts (Figure 9B). To identify forced expiratory volume (FEV) in ferrets, we utilized expiratory volume measured at 0.4 seconds (FEV_0.4_), which has been established for ferrets as analogous to human FEV_1_ (32). The percentage predicated FEV_0.4_ for *CFTR*-F508del ferrets was significantly (*P* = 0.0034 and *P* = 0.022) reduced when compared with the larger WT control cohort and age- and sex-matched WT controls, respectively (Figure 9C). The percentage predicated forced vital capacity (FVC) was also significantly reduced (*P* = 0.0061) in *CFTR*-F508del ferrets, but only in comparison with the age- and sex-matched WT cohort (Figure 9D). The FEV_0.4_/FVC ratio was significantly elevated in *CFTR*-F508del ferrets, but only in comparison with the larger WT control cohort (Figure 9E; *P* = 0.0023). This restrictive lung disease pattern of decreased FEV_0.4_ and FVC with an increased FEV/FVC ratio can occur with air trapping and is occasionally observed in people with CF with small airway disease, rather than the more typical obstructive pattern.

One of the clinical features of mucus accumulation, air trapping, and scarred tissue in the lungs is increased stiffness, leading to reduced lung compliance as commonly seen in a restrictive lung phenotype. We measured the lung compliance of *CFTR*-F508del and WT ferrets by constructing pressure-volume loop (PV-loop) curves (Figure 9F) and noted that both female and male *CFTR*-F508del ferrets were shifted downward (*P* < 0.0001) from the paired WT control group. Comparing the measured quasistatic compliance (Cst) (Figure 9G) and dynamic compliance (Crs) (Figure 9H), we found that both measures of lung compliance were significantly lower in *CFTR*-508del ferrets. This is consistent with reduced IC (Figure 9, A and B) and increased airway resistance (Figure 9I), respectively. Elevated resistance of the respiratory system (Rrs) in *CFTR*-F508del ferrets (Figure 9I) is consistent with our findings of mucus and airway inflammation that would impede airflow. Taken together, our results show that *CFTR-*F508del ferrets displayed components of both obstructive (reduced IC and FEV) and restrictive (reduced quasi-static compliance and increased FEV_0.4_/FVC) lung disease, which may also reflect air trapping and increased residual volume.

## Discussion

Here, we present the characterization of a *CFTR*-F508del ferret model that develops multiorgan disease like people with CF and is partially responsive to first-generation CFTR combination modulator therapy (LUM/IVA). Important aspects of this study include the efficacy of in utero LUM/IVA treatment in preventing MI and improving postnatal survival. In the absence of CFTR modulator therapy, *CFTR*-F508del ferrets spontaneously developed bacterial lung infections and changes in pulmonary mechanics, which may be useful for the field in developing genetic therapies in the ferret model, with surrogate efficacy endpoints similar to those used in CF clinical trials.

Previous studies evaluating recombinant CFTR-F508del protein have demonstrated species-specific differences in processing, with a ranked severity of human > ferret > pig > mouse (28, 33, 34). Studies in cell lines have shown that approximately twice as much fCFTR-F508del protein is processed to the plasma membrane than the human CFTR-F508del counterpart (28). The majority of findings in *CFTR*-F508del ferrets support residual function and processing of the fCFTR-F508del protein to the plasma membrane. First, the incidence of MI in untreated *CFTR*-F508del ferrets (60%) was less than that observed in *CFTR*-KO ferrets (~80%) (21, 29). Second, newborn *CFTR*-F508del organoids demonstrated a significant swelling response to IVA alone at 0.128 μM forskolin, whereas this was not observed in adult *CFTR*-F508del organoids. Thus, the fetal intestine may have more CFTR-F508del at the membrane due to higher expression or improved processing and this may be responsible for the reduced incidence of MI and improved postnatal survival of *CFTR*-F508del ferrets treated with IVA alone. Third, *CFTR*-F508del airway cultures produced cAMP-inducible Cl^–^ currents that were approximately 10% of that of WT cultures, which is approximately double that of human F508del-homozygous airway cultures (27).

Phenotypic heterogeneity in the pulmonary and nutritional status of F508del-homozygous individuals has been shown to correlate with intestinal organoid FIS (35). Thus, the mechanism responsible for the observed differences in f*CFTR*-F508del processing and residual CFTR function may underlie clinical heterogeneity in F508del-homozygous people with CF. This raises the important question of whether small changes in CFTR-F508del residual processing and function have a long-term impact on disease progression. The *CFTR*-F508del ferret model may have utility in addressing how much CFTR activity is required to slow disease progression in various CF-affected organs. For example, mucus obstruction in the lung of *CFTR*-F508del ferrets removed from modulators was generally less severe than in *CFTR*-KO (36) and *CFTR*-G551D ferrets (21), despite bacterial colonization of the lung in all 3 genotypes. By contrast, pancreatic disease was uniformly progressive in *CFTR*-F508del ferrets removed from modulators and similar to *CFTR*-KO (31) and *CFTR*-G551D ferrets (21), suggesting that residual fCFTR-F508del activity was insufficient to slow pancreatic disease. Furthermore, in utero IVA monotherapy in *CFTR*-F508del ferrets demonstrated a low level of protection from MI, but failed to slow postnatal exocrine pancreatic disease, and protection of the exocrine pancreas was only observed in a subset of LUM/IVA–treated *CFTR*-F508del ferrets. Cumulatively, these findings suggest that the CFTR activity threshold for protection from disease is highest in the pancreas. Subtle differences in the expression of modifier genes that impact CFTR-F508del processing and function may underlie this phenotypic variability in both humans and ferrets with CF (37, 38).

Currently, there are no CFTR modulators approved for the treatment of CF in utero or during the first month of life. However, there are a rising number of pregnancies in CF women and this is leading to a growing number of case studies where CF fetuses and infants are being exposed to CFTR modulators during this developmental period (16, 17). For example, a pregnant F508del homozygous CF mother, who elected to remain on ETI therapy during pregnancy and nursing, gave birth to a CF infant who tested negative in newborn screening (39). This infant remained pancreatic sufficient for multiple months after birth, with the only abnormality being elevated sweat chloride levels. These types of case studies, together with our findings in the *CFTR*-F508del ferrets, highlight the importance of early treatment in preventing the onset of CF disease manifestations.

One of the more impactful findings in our study was the clear demonstration of altered pulmonary mechanics in *CFTR*-F508del ferrets using the custom-designed flexiVent system. Percentage predicted FEV_1_ is the most commonly studied marker for progression of lung disease patients with CF, and the equivalent measure (FEV_0.4_) in *CFTR*-F508del ferrets was also significantly lower than WT controls. A current limitation of PFTs in ferrets is limited WT baseline data (68 animals with 160 measurements) on which we calculated percentage predicted IC, FEV, and FVC values. An additional challenging feature was the need to perform measurements similarly to full forced expiratory maneuvers in infants (40). However, our statistical comparisons did incorporate age, sex, and genotype as independent variables. The increased percentage FEV_0.4_/FVC in the *CFTR*-F508del ferrets was unanticipated, since this ratio is most often reduced or normal in CF adolescents and adults, indicating obstructive lung disease (reduced FEV) with a reduction in FVC (41). However, others have proposed that reversible restrictive processes involving small airways disease can alter CF pulmonary mechanics and reduce percentage FEV/FVC (42). Given that ferret FEV and FVC measurements were obtained using a custom-built negative pressure forced exhalation chamber, we also cannot rule out that the increased FEV_0.4_/FVC in *CFTR*-F508del ferrets is a feature of these ferret-specific pulmonary maneuvers. Regardless of this FEV_0.4_/FVC difference in clinical correlations to the *CFTR*-F508del ferret model, PFT endpoints may serve as useful endpoints for therapeutic studies in the model, and with the exception of the FEV_0.4_/FVC ratio, these PFT endpoints reasonably correlated with disease progression in people with CF.

This study has several limitations. First, this study evaluating LUM/IVA therapy in *CFTR*-F508del ferrets was initiated prior to FDA approval of ETI. Due to the limited availability of ELX, in both quantity and powdered form required for formulation, we have been unable to evaluate this next-generation CFTR corrector in animals. Second, as discussed above, our limited normative lung function data have likely affected PFT metric comparisons between cohorts. Third, our ferret cohorts were rather small and variable severity of disease progression prevented longitudinal PFT measures, which are most informative in studying disease progression.

In summary, we describe a *CFTR*-F508del ferret model that is partially responsive to first-generation combination CFTR modulator therapy and develops multiorgan disease in the absence of modulators. These studies also complement previous work evaluating processing of recombinant fCFTR-F508del protein by demonstrating that trafficking of the mutant protein in primary cells is responsive to LUM, ELX, and TEZ. Thus, the *CFTR*-F508del ferret model may have utility for studying mechanisms of CFTR-F508del biosynthesis in vivo, disease pathogenesis, and for assessing therapeutics that target lung and pancreatic diseases.

## Methods

### Sex as a biological variable.

Our study examined male and female ferrets and accounted for this covariate in our statistical analyses. We observed sex-dependent differences in respiratory mechanic parameters. Therefore, we used sex-matched ferrets for the paired-analysis tests.

### Generation of CFTR-F508del ferret model using CRISPR/Cas9-mediated homologous recombination.

A CRISPR/Cas9–recognized sequence (ATCAAAGAAAACATCAT//CTT-TGG) was chosen using the sgRNA designing tool CRISPOR (http://crispor.tefor.net), in which the CRISPR cleavage site is immediately before the F508 codon in exon 11 of the *CFTR* locus. We synthesized this gRNA through T7 RNA polymerase–mediated in vitro transcription from a DNA template containing a T7 promoter and a 20-nt recognition sequence, followed by the *Streptococcus* *pyogenes* sgRNA scaffold, which was synthesized as a gBLOCK by Integrated DNA Technologies (IDT), and functionally validated using an in vitro Cas9 cleavage assay. Cas9 mRNA (5meC, Ψ, catalog L-6125) was purchased from TriLink. A mutagenesis DNA donor fragment, which lacked the F508 codon, was a 190-nt single-stranded oligonucleotide synthesized by IDT. Zygotes were collected as previously described (43). The mixture containing a final concentration of 200 ng/μL Cas9 mRNA, 100 ng/μL gRNA, and 20 ng/μL donor single-stranded oligonucleotide was microinjected (Femptojet, Eppendorf) into the ferret zygote cytoplasm. Injected embryos were cultured overnight in TCM-199 plus 10% FCS media to the 2-cell stage prior to being transferred into primipara pseudopregnant jills. The kits were naturally delivered after 42 days of gestation. To identify the mutants, DNA was extracted from kit tail clips for PCR using a primer set (Forward: TGATGATTATGGGAGAGTTGGAGCC and Reverse: GCATGCATATAAGTGTCCACTGAGG) that amplified DNA a product of 530 bp (F508del) or 533 bp (WT). PCR products were sequenced and used in TIDE analysis (https://tide-calculator.nki.nl) to identify kits harboring the mutation. The PCR products containing targeted mutations were also cloned into pCR4Blunt via Topo Cloning and Sanger sequencing was used to confirm the F508del allele.

### Ferret rearing.

LUM/IVA (15 mg/kg body weight each, twice daily, formulated in medium-chain triglyceride [MCT] oil) were administered orally to pregnant jills, starting on day 28 of gestation. Newborn kits were gavaged daily with LUM/IVA (5 mg/kg, twice daily, each formulated in MCT oil). The dose was increased to 10 mg/kg twice daily at 10 days of age and increased to 15 mg/kg twice daily at 30 days of age and maintained at that dose thereafter. Animals were later withdrawn from LUM/IVA at various ages to evaluate the disease progression. In a subset of animals, only IVA was used to modulate CFTR.

### Genotyping of newborn F508del ferrets.

To facilitate more rapid genotyping that did not require sequencing, we developed a Surveyor assay. PCR products were generated with the above primer set and 2 independent Surveyor assays were performed to distinguish WT and F508del alleles. The PCR products were used directly for the first Surveyor assay, which discriminated between the heterozygous genotype (F508del/WT) and homozygous genotypes (WT/WT and F508del/F508del). To distinguish between homozygous genotypes, a second Surveyor assay was performed after mixing the remaining PCR product with an equal amount of PCR product from a known WT ferret. Approximately 100 ng of genomic DNA was used for the PCR reaction with the following settings: Step 1, 95°C for 2 minutes; Step 2, 95°C for 30 seconds; Step 3, 58°C for 30 seconds; Step 4, 68°C for 40 seconds (Steps 2, 3, and 4: 38 cycles), and final extension at 68°C for 15 minutes. PCR products (10 μL either neat or mixed with WT) were then denatured at 95°C for 10 minutes, and re-annealed at 93°C for 1 second, 91°C for 1 second, 89°C for 1 second, 87°C for 1 second, and 85°C for 1 second, followed by decreasing 1°C per second down to 25°C. These PCR products were incubated with 2.5 U (1 μL volume) T7 Endonuclease I (New England BioLabs) for 1 hour at 37°C. The products from Surveyor assays were electrophoresed in a 1.5% agarose gel. A detailed standard operating procedure for genotyping can be found in the Supplemental Material.

### Necropsy and histopathology.

Lung, pancreas, and gallbladder were fixed in 10% neutral buffered formalin (NBF), embedded in paraffin, and sectioned at 4 μm. Lungs were either inflated with 10% NBF or fixed without insufflation. Paraffin sections were stained with H&E, PAS, Alcian blue, Gram, and/or Masson’s trichrome stain and evaluated with bright-field light microscopy.

### Quantification of fCFTR mRNA.

Total ferret RNA was extracted using the Qiagen RNeasy plus kit from intestinal organoids, newborn trachea, proximal intestine, and lung accessory lobe and converted to cDNA using the ABI high-capacity cDNA synthesis kit for RT-qPCR. The sequences of the primer set and the probe specific for a 124-bp amplicon of the f*CFTR* were as follows: Forward, TGGCTTGGAAATCAGTGAGG; Reverse, CTTGTGGATAGTAACATATCGGAGG; and Probe, 6FAM-ACTGGTGGTATGCTTTCAACATCGTCA. The sequences of the primer set and the probe specific for a 137-bp amplicon of f*GAPDH* were as follows: Forward, CAACTTTGGCATTGTGGAGG; Reverse, CAGTGGAAGCAGGGATGATG; and Probe, 6FAM-CAGTGATGGCATGGACGGTGG.

### CFTR protein Western blots.

Ferret intestinal organoids were solubilized and lysed in RIPA buffer (Thermo Fisher Scientific) with a protease inhibitor. The lysed organoids were centrifuged for 5 minutes at 21,130*g* and the supernatant was used for the subsequent steps. The concentration of the supernatant was determined by a BCA assay (Thermo Fisher Scientific). Organoid lysate (10 μg) was incubated with 6× loading dye at 37°C for 5 minutes. Samples were loaded on a NuPage 3%–8% Tris-acetate protein gel (Thermo Fisher Scientific) and run for 2.5 hours at 90 V. Proteins were transferred to a nitrocellulose blotting membrane (GE Healthcare) for 60 minutes at 400 mA. The membrane was blocked using a blocking buffer (5% dry milk in PBS with 0.1% Tween 20, PBST) for 1 hour, followed by incubation with an anti-CFTR (CFTR 596, UNC CFTR Antibody Distribution Program; 1:1000 dilution in the blocking buffer) and anti–β-tubulin antibody (Abcam, catalog ab41489; 1:1000 dilution) overnight at 4°C. The membrane was washed 3 times with PBST and then incubated with a donkey secondary anti-mouse HRP-conjugated antibody (Jackson ImmunoResearch; 1:2000) for 1 hour. After the incubation, the membrane was washed extensively with PBST, and the HRP substrate was developed using SuperSignal West Femto Maximum Sensitivity Substrate (Thermo Fisher Scientific) and imaged on the iBright CL-1000 imaging system (Thermo Fisher Scientific). The Western blotting of ALI cultures was done after lysing the differentiated airway cells in RIPA buffer with a protease inhibitor. Cell lysates were briefly sonicated and centrifuged at 21,130*g* for 5 minutes and the supernatant was used for the Western blotting. The intensities of the B and C bands were calculated with Fiji image-processing software (44) and normalized to the band intensity of β-tubulin.

### Generation of intestinal organoid cultures.

Ferret intestinal organoids were isolated from newborn and adult ferret intestines. The intestines were dissected, minced, and washed in cold PBS by pipetting up and down to remove villi. The washed tissues were incubated in Accutase Cell Detachment Solution (Innovative Cell Technologies, Inc.) at room temperature for 20 minutes with rocking to help release the crypts. The crypts were isolated by mechanical disruption of the intestines in PBS with 0.1% BSA using a pipettor. The crypts were separated from the main tissue using a 100-μm filter, centrifuged at 290*g* for 5 minutes, and the pellets were washed with F12 supplemented with 1% HEPES, 1% GlutaMAX (Thermo Fisher Scientific), and 1% penicillin/streptomycin (P/S). The pellet was mixed with cooled IntestiCult Organoid Growth media (StemCell Technologies) and Matrigel (Corning, Inc) (50:50) and seeded on 24-well plates warmed up to 37°C. Organoids typically formed in 24 to 36 hours and were then passaged into new 24-well plates before seeding in 96-well plates for imaging.

### FIS assays.

Ferret intestinal organoids plated in a 96-well plate were used for imaging. Prior to measurement, organoids were labeled with a calcein green AM (Thermo Fisher Scientific) dye for 30 minutes at a final concentration of 1 μM. FIS of WT/WT, KO/KO, and F508del/F508del organoids was assessed by measuring the response to various concentrations of forskolin (0.02, 0.128, 0.8, and 5 μM final concentration) in the presence of vehicle (DMSO), potentiator (IVA, 3 μM), corrector (LUM, 3 μM), or both LUM/IVA (each 3 μM) over a fixed 60-minute period with images captured at 10-minute intervals (total 7 time points). Zeiss confocal software (Zen Blue, edition 2.3) was used to calculate the area (μm^2^) as previously described (45).

### Airway cell isolation, culture, and Isc measurements on polarized ferret tracheal airway epithelium.

Airway basal cells were generated according to a published method (46), with minor modifications. Briefly, 1- to 2-month-old ferret tracheae were first washed with a strong antibiotic solution (100 μg/mL Primaxin 100 [InvivoGen], 1 μg/mL amphotericin B, 100 μg/mL ceftazidime, 125 μg/mL gentamicin, and 2% P/S). After several washes, the tracheae were transferred into a protease solution (1 mg/mL pronase) in the same antibiotic media and gently rocked overnight at 4°C. The next day, tracheae were minced into smaller pieces and the tissue was passed through a 100-μm filter. The filtrate containing the detached cells was collected and centrifuged at 500*g* for 5 minutes. The cells were plated on plastic in PneumaCult-Ex Plus media (StemCell Technologies) containing 2× amphotericin B and Primaxin 100 to prevent microbial and mycoplasmic growth, respectively. All culture plates used for cell expansion procedure were precoated with conditioned media obtained from 804G cells to support epithelial cell attachment. After 2 days of culturing, cells were passaged to a larger flask. Once the cells neared confluence, they were detached from the flask using a mild dissociation reagent (Accutase, Innovative Cell Technologies). Cells (1 × 10^5^) were seeded per permeable Transwell (6.5 mm, 0.4 μm polyester, precoated with collagen; Costar) in a 24-well plate. Cells were cultured in PneumaCult-Ex Plus medium for 2 days for the expansion, and subsequently in PneumaCult-ALI Medium (StemCell Techologies) for at least 2 weeks for differentiation. The resistance of the cells was checked periodically to ensure that it reached at least 1000 Ω prior to Isc experiments. F508del cultures were prestimulated overnight with either 3 μM LUM (in DMSO) or an equal volume of DMSO (vehicle control). The following day, transepithelial Isc was measured in the presence of an equal amount of DMSO (vehicle control) and LUM/IVA in the apical side of an Ussing chamber, while amiloride (100 μM), DIDS (100 μM), IBMX (100 μM)/forskolin (10 μM), and GlyH101 (50 μM) (all from MilliporeSigma) were added sequentially to the apical chamber, as previously described in detail for airway ALI cultures (46). A symmetrical buffer system was used in each Ussing chamber for measuring both the chloride and bicarbonate currents. The chloride buffer consisted of 135 mM NaCl, 2.4 mM K_2_HPO_4_, 0.6 mM KH_2_PO_4_, 1.2 mM CaCl_2_, 1.2 mM MgCl_2_, 10 mM dextrose, and 5 mM HEPES (pH 7.4) gassed with air. The bicarbonate buffer consisted of 118.9 mM sodium gluconate, 25 mM NaHCO_3_, 2.4 mM K_2_HPO_4_, 0.6 mM KH_2_PO_4_, 5 mM calcium gluconate, 1 mM magnesium gluconate, and 5 mM dextrose (pH 7.4) gassed with 5% CO_2_.

### PFTs.

Lung function tests were performed on age- and sex-matched *CFTR*-F508del and WT ferrets using a forced oscillation technique (flexiVent, SCIREQ), as previously described (32). The plethysmograph chamber and Aeroneb components were calibrated for each endotracheal tube before sedating the ferrets according to the manufacturer’s protocol. Ferrets were first sedated using a xylazine (0.25–1.5 mg/kg) and ketamine (5–25 mg/kg) mixture after completion of the flexiVent system calibration. The same endotracheal tube used for calibration was also used for the experimental ferret and prior to endotracheal tube placement, the ferret was maintained on 5% isoflurane using a nose cone to maintain a passive subject. The ferret was then intubated, transferred to the plethysmograph chamber, and the isoflurane was reduced to 2% after the ferret was passively breathing with the flexiVent. The vitals of animals were checked every 10 minutes before, during, and after the procedure. Two different scripts were used for PFT to help account for differences in ferret size by using a 2-stroke maneuver to measure the IC. For small ferrets (up to 4 months of age or less than 800 g of weight), a “small ferret” script was used, which consisted of a single stroke of volume replacement for calculating the IC. For ferrets older than 4 months or greater than 800 g, a “large ferret” script was used, which allowed a multistroke maneuver to collect IC data sequentially. Overall, a total of 6 perturbations were performed for each ferret. Before each perturbation, each ferret was ventilated with the flexiVent system for 2 to 3 minutes to ensure they were completely passive for the experiment. Each perturbation included (a) deep inflation to measure IC; (b) SnapShot-60 (a maneuver in the SCIREQ flexiVent system to study the lung under conditions of tidal breathing) for dynamic compliance (Crs), elastance (Ers), and resistance (Rrs); (c) PV-loop to calculate quasistatic compliance (Cst); and (d) negative pressure forced exhalation to collect expiratory volumes (FVC, FEV_0.4_). For all perturbations, parameters were taken into account only if the coefficient of determination was greater than 0.9. Two detailed standard operating procedures for using the flexiVent and processing data can be found in the Supplemental Material.

### Statistics.

Statistical analysis was performed using Prism 8 software (GraphPad). Data are expressed as mean ± SEM of *n* independent measurements. When only 2 data sets were compared, a paired, 2-tailed Student’s *t* test was used for statistical comparison (Western blot quantification). Comparison of MI frequencies and postnatal survival used Fisher’s exact test. For comparison of 3 or more data sets, the statistical results were calculated using 1-way ANOVA with Bonferroni post hoc test (Isc) or Tukey’s post hoc test (bacterial CFU), or 2-way ANOVA with Bonferroni’s post hoc test (organoid swelling assays). Given the large sexual dimorphism in the size of ferrets, normalizing PFT endpoints with body length was justified in our previous ferret studies (32) because of the strong correlation between length and PFT endpoints (IC, FEV, FVC) (Supplemental Figure 4). To obtain more accurate PFT metrics, we performed linear regression analysis for length versus IC, FEV, and FVC from the large cohort of WT ferrets. These regression equations for each sex were then used to generate a predicted PFT value for each animal based on its length. The actual measured value for each animal is expressed as a percentage of that predicted value. For the analysis of the PFT data, statistical comparisons used a mixed-effects model, which accounted for repeat measures in a subset of animals, using R software (http://www.R-project.org/) and the lmer function within the R package lme4. For comparison of age- and sex-matched *CFTR*-F508del cohort to the WT cohort (*n* = 14 measurements), a mixed-effects model was used, in which ferret ID and the pairing of the ferrets were random effects and genotype was a fixed effect. For the statistical comparison of *CFTR*-F508del cohort (*n* = 14 measurements) to a larger WT ferret cohort (*n* = 160 measurements), the mixed-effects model included ferret ID (accounting for repeat measure), age, sex, and genotype as independent variables. The PV-loop data were analyzed by creating a new continuous pressure variable fitting by quadratic regression. A mixed-effects model was then applied using the genotype and the square of the pressure variable as fixed effects and ferret ID as a random effect. In all statistical analyses, *P* values less than 0.05 were deemed significant.

### Study approval.

All animal care and experimentation were performed according to a protocol (no. 1071945) approved by the University of Iowa’s Institutional Animal Care and Use Committee.

### Data availability.

All raw data underlying the results of this study can be found in the supplemental Supporting Data Values file.

## Author contributions

IAE conceived and designed the study, conducted experiments, acquired and assembled data, analyzed and interpreted data, and wrote the manuscript. BL, ARV, LG, Yan Zhang, Yulong Zhang, Y Yi, Y Yang, ZF, SYP, AD, TJL, AS, HM, LQ, PW, GL, and AL acquired, analyzed, and interpreted data. ZY generated and genetically characterized the ferret model. XS generated and genetically characterized the ferret model, interpreted data, and wrote the manuscript. DKM and KNGC interpreted histopathological results, acquired data collection, and wrote the manuscript. DHL and BHR interpreted data and wrote the manuscript. KW statistically analyzed data and wrote the manuscript. DJB and JFE conceived and designed the study, analyzed and interpreted data, wrote the manuscript, and approved the final manuscript version.

## Supplementary Material

Unedited blot and gel images

Supporting data values

Supplemental data

## Version 1 04/22/2024

Electronic publication
