# Supplementary figures and images for: In utero and postnatal ivacaftor/lumacaftor therapy rescues multiorgan disease in CFTR-F508del ferrets
Source: JCI Insight. 2024 Apr 22;9(8):e157229. doi: 10.1172/jci.insight.157229 (PMC11141870; doi:10.1172/jci.insight.157229)

Figure 2A

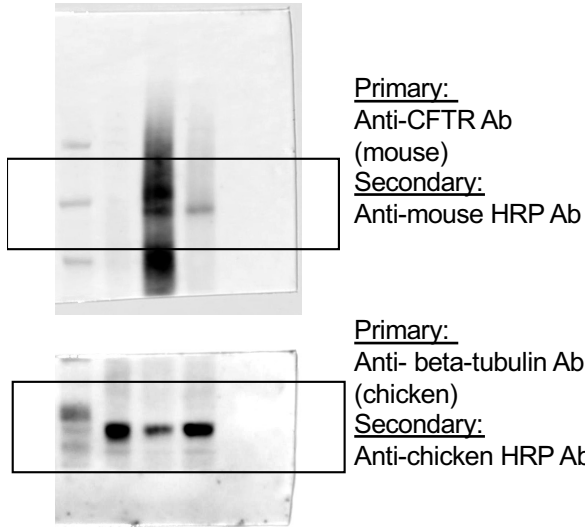

Figure 2B

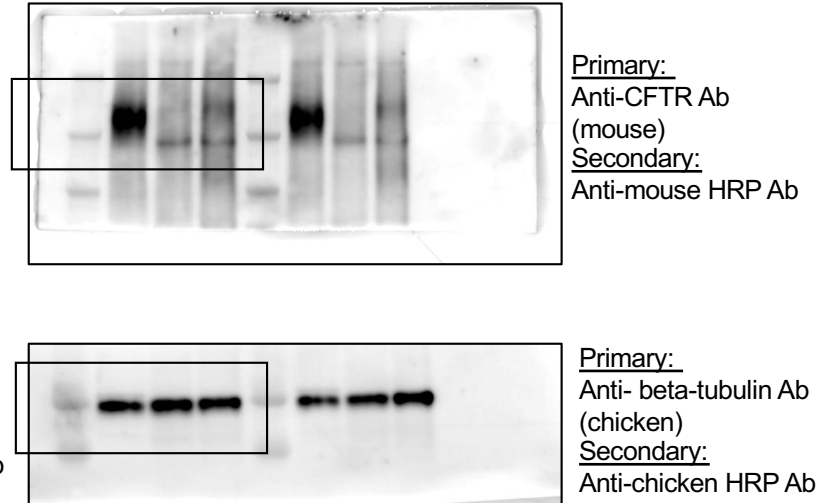

Figure 2C

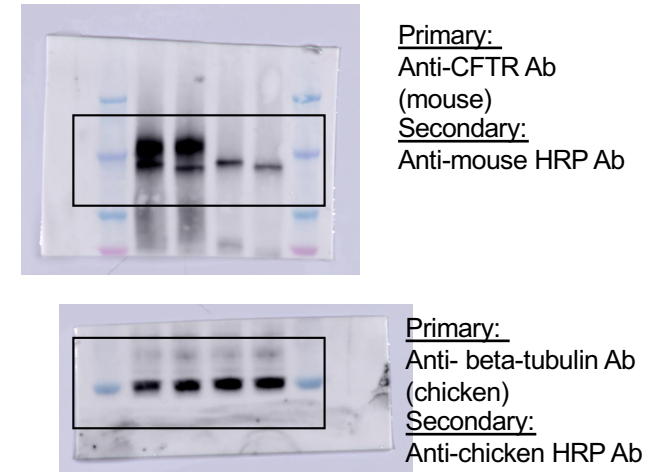

Figure 2D

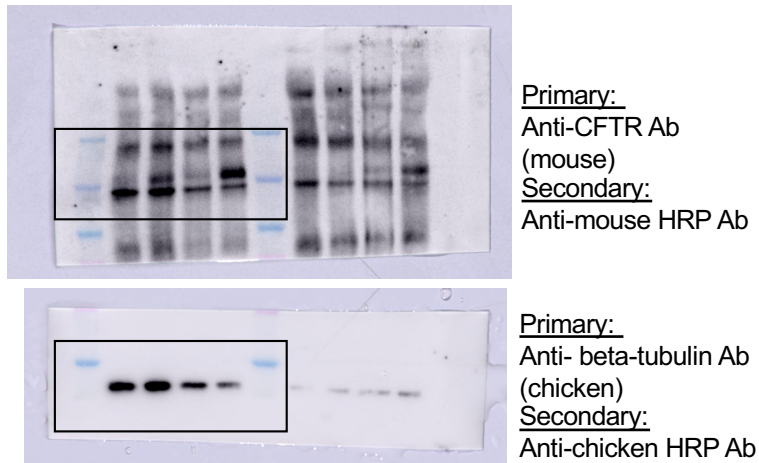

Figure 2E

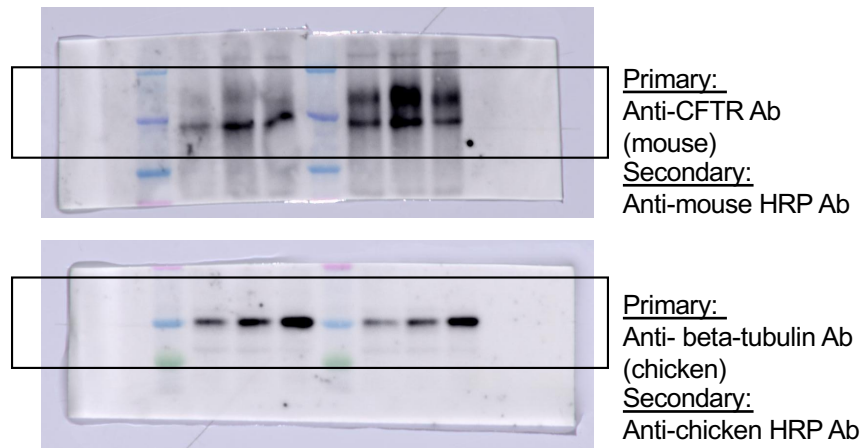

Supplement: Unedited blot and gel images [file jciinsight-9-157229-s097.pdf]
