# Supplementary material for: In utero and postnatal ivacaftor/lumacaftor therapy rescues multiorgan disease in CFTR-F508del ferrets
Source: JCI Insight. 2024 Apr 22;9(8):e157229. doi: 10.1172/jci.insight.157229 (PMC11141870; doi:10.1172/jci.insight.157229)
Supplement: Supplemental data [file jciinsight-9-157229-s099.pdf]

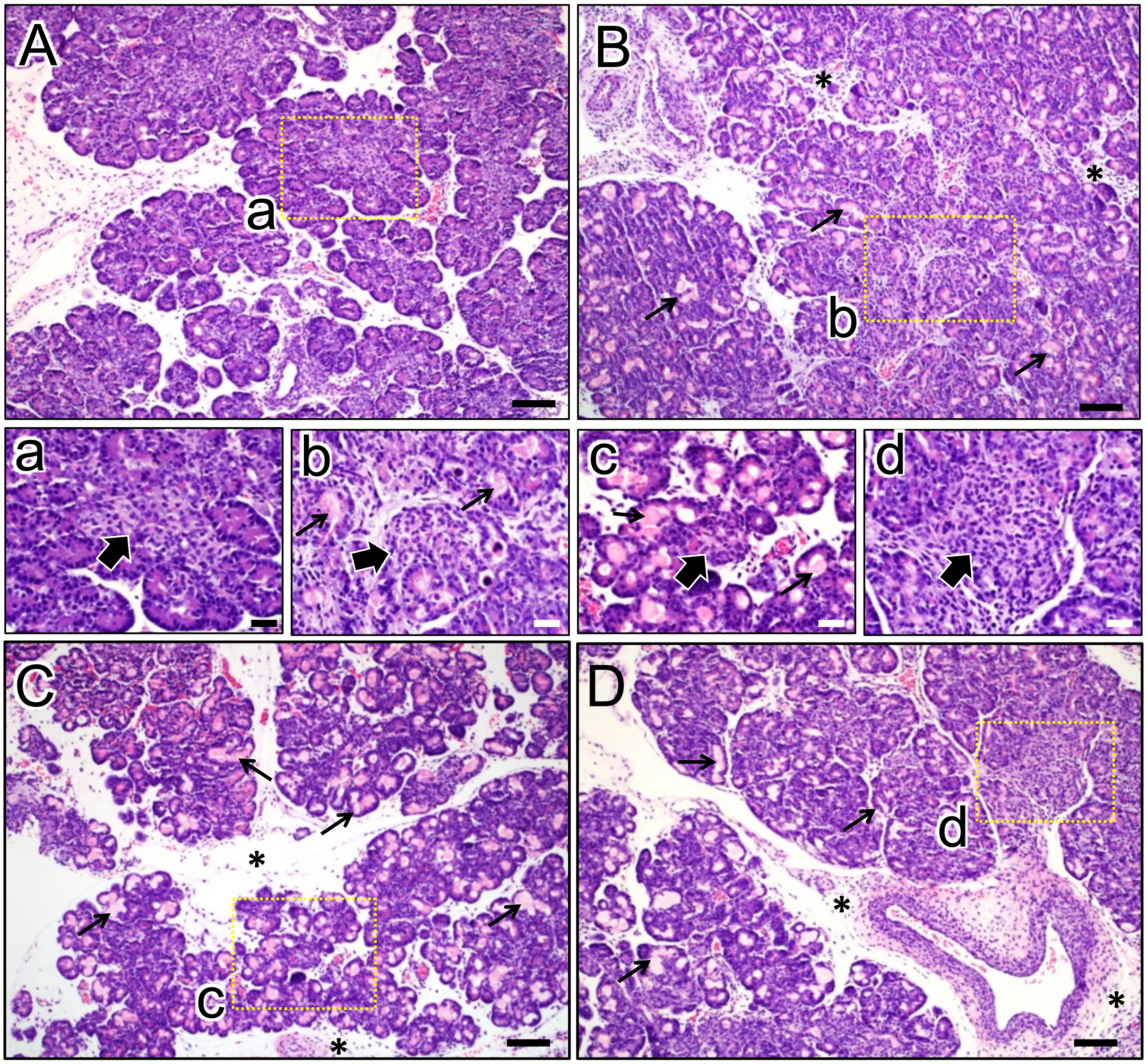

**Supplemental Figure S1. Pancreatic pathology in newborn WT and *CFTR*-F508del kits. (A)** Representative H&E-stained section of the pancreas from a newborn WT ferret. (**B-D)** H&E-stained pancreatic sections from three independent newborn homozygous *CFTR*-F508del ferrets not treated with CFTR modulators *in utero*. Note the edematous pancreatic interstitial tissue (*) and mild to moderate acinar dilation with homogenous eosinophilic secretory material (thin arrows). Islet morphology appears unaffected in *CFTR*-F508del pancreata (insets marked by large arrows). **(a-d)** Higher magnifications of boxed regions in the main panels. **(A-D)** Micron bars = 100 μm; **(a-d)** Micron bars = 40 μm.

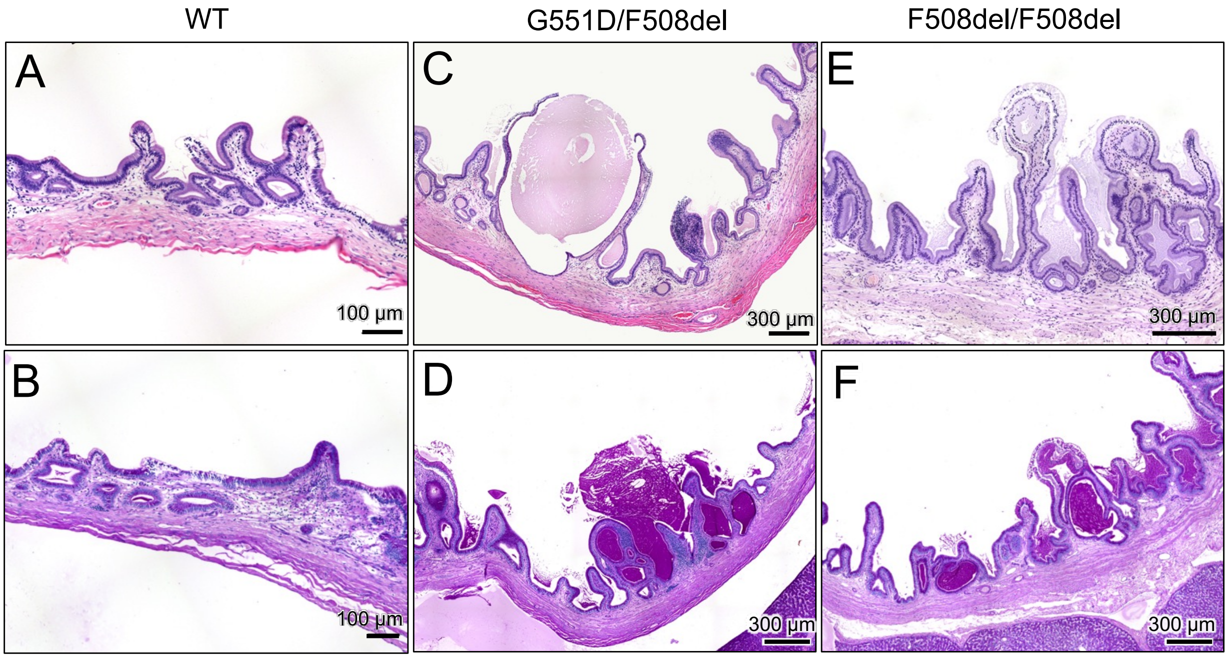


**Supplemental Figure S2. Gallbladder pathology in adult *CFTR*-F508del ferrets. (A-F)** Adult gallbladders from **(A, B)** WT, **(C, D)** G551D/F508del compound heterozygote, and **(E, F)** F508del/F508del homozygous ferrets stained with **(A, C, E)** H&E or **(B, D, F)** PAS. The two adult *CFTR*-F508del ferrets were reared on only IVA until **(C, D)** 20 days or **(E, F)** 117 days of age and then euthanized at 32 or 16 months of age, respectively. One ferret **(C, D)** was a compound heterozygote of the *CFTR*-F508del/G551D genotype and the other **(E, F)** had a homozygous *CFTR*-F508del/F508del genotype.

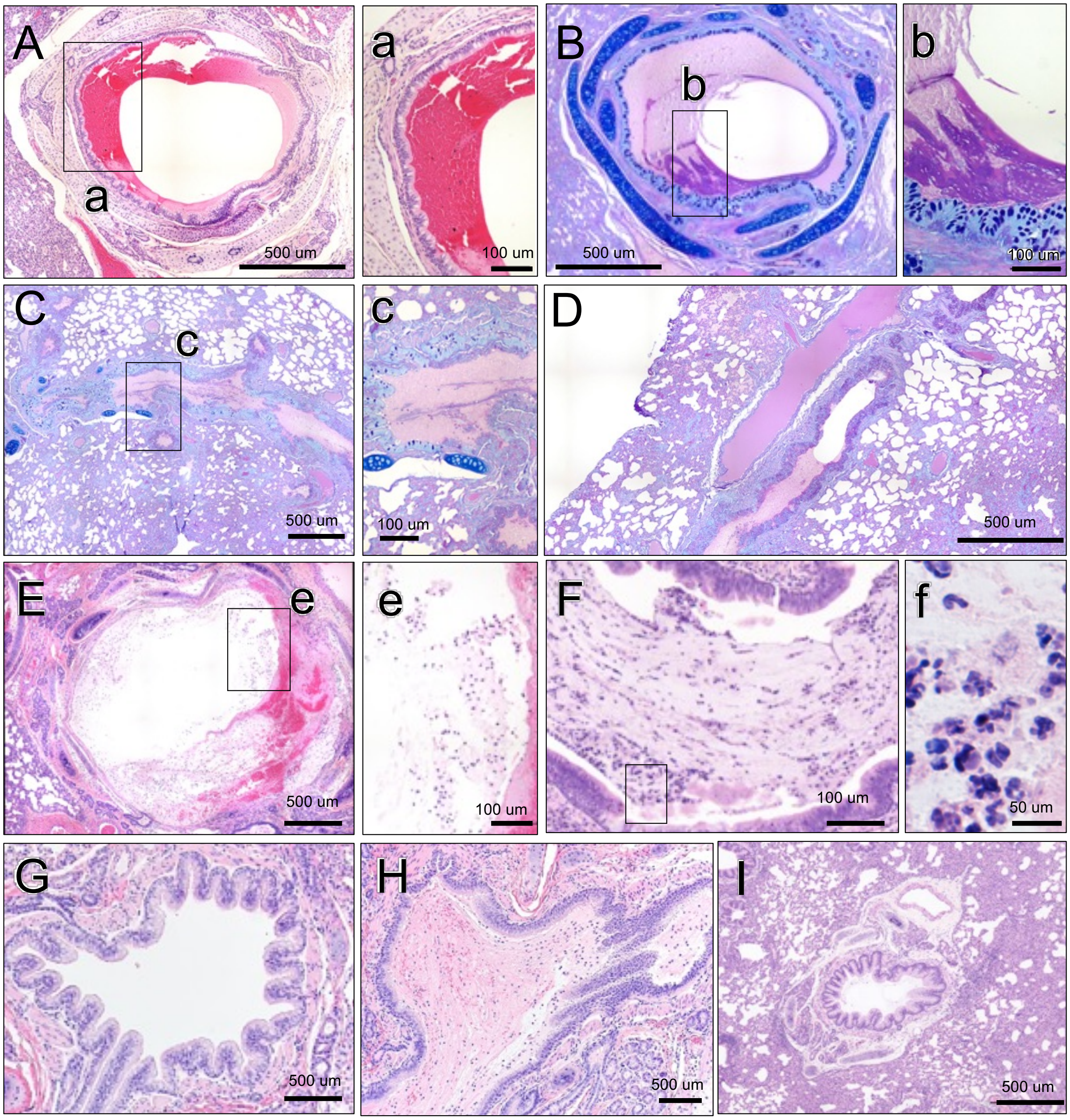


**Supplementary Figure S3. Lung phenotype of *CFTR*-F508del ferrets that reached terminal euthanasia endpoint upon cessation of LUM/IVA.** H&E **(A)** and PAS **(B)** stained sections of large airways from RUL of a *CFTR*-F508del ferret that died at the age of 101 days showing accumulation of blood and mucus. Alcian blue staining of small airways from the same ferret is shown in **C and D**. H&E stained sections from left lower lobe **(E)** and right middle lobe (RML) **(F)** of 113 and 136 days old *CFTR*-F508del ferrets that show epithelial necrosis and **(E, e)** and accumulation of macrophages **(F, f)**. Other small airways are shown in **G, H,** and **I**. Micron bars: (A-I): 500 µm; (a, b, c, e): 100 µm, f: 50 µm.

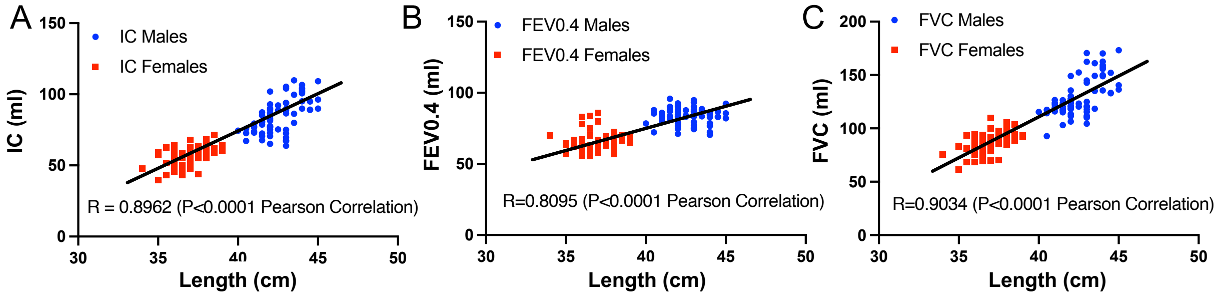


**Supplementary Figure S4. Correlation between ferret body length and PFT measures**. **(A-C)** Body length of WT male (blue) and female (red) ferrets blotted against IC **(A)**, FEV0.4 **(B)**, and FVC **(C).** Correlation coefficient (R) and P-values shown were determined by Pearson correlation test.

# *CFTR*-F508del Genotyping SOP

- Solutions
  - Invitrogen Accuprime PFX Supermix (Catalog No: 12344040)
  - OLI009 CFTR-DF FF1 (TGA-TGA-TTA-TGG-GAG-AGT-TGG-AGC-C)
  - OLI010 CFTR-DF Intron11-R3 (GCA-TGC-ATA-TAA-GTG-TCC-ACT-GAG-G)
  - New England Biolabs NEBuffer 2 (Catalog No. NEB #B7202)
  - New England Biolabs T7 Endonuclease I(Catalog No. NEB #E3321)
  - 1X TAE buffer
  - Agarose
  - Ethidium Bromide (10mg/mL)
  - Invitron TrackIt Cyan/Orange Loading Buffer (Catalog No. 10482-028)
  - TrackIt 1kb Plus DNA Ladder (Catalog No. 10488085)
- Equipment
  - Thermocycler
  - 2ml tube
  - Various Pipettes and associated filter tips

1. Create Mastermix 1

| Component | Amount(µl)/Sample |
|---|---|
| 10x HiFi Buffer | 2.5 |
| 50mM MgSO4 | 2 |
| 25mM dNTP | 0.3 |
| dd Water | 18 |
| Platinum Taq HiFi Polymerase | 0.25 |
| OLI009 CFTR-DF FF1 (10mM) | 1.25 |
| OLI010 CFTR-DF Intron11-R3 (10mM) | 1.25 |

2. Create Mastermix 2

| Component | Amount(µl)/Sample |
|---|---|
| 10x HiFi Buffer | 2.5 |
| 50mM MgSO4 | 2 |
| 25mM dNTP | 0.3 |
| dd Water | 18 |
| Platinum Taq HiFi Polymerase | 0.25 |
| OLI009 CFTR-DF FF1 (10mM) | 1.25 |
| OLI010 CFTR-DF Intron11-R3 (10mM) | 1.25 |
| WT DNA Sample | 0.5 |

3. Digest tissue samples according to the Qiagen DNeasy Blood and Tissue Kit protocol.
   a. NOTE: Elute with 50µl AE buffer at final step
4. Aliquot 1 µl of digested sample into labeled PCR Tubes
5. Using a different pipette tip for each tube, aliquot 24 µl of mastermix into each tube
6. Under the Ferret Genotyping Folder, run the CFTR-DF Thermocycler protocol

| Step | Temperature | Time (min) |
|---|---|---|
| Step 1 | 95˚C | 02:00 |
| Step 2 | 95˚C | 00:30 |
| Step 3 | 58˚C | 00:30 |
| Step 4 | 68˚C | 00:40 |
| Step 5 | 68˚C | 15:00 |
| Step 6 | 10˚C | Hold |

Repeat step 2-step4 38 times.

7. Create NEBuffer 2 Secondary mix

| Component | Amount(µl)/Sample |
|---|---|
| DI H2O | 1.5µl/Sample |
| NE Buffer 2 | 1.5 µl/Sample |

8. Add 10 ul of PCR product to new microcentrifuge tubes.
9. Add 3 ul of the NE 2 Secondary Buffer mix to each tube. Vortex and spin down.
10. Under the Ferret Genotyping Folder, run the Denature Annealing Thermocycler Procedure

| Step | Temperature | Time(min) |
|---|---|---|
| Step 1 | 95˚C | 10:00 |
| Step 2 | 93˚C | 00:01 |
| Step 3 | 91˚C | 00:01 |
| Step 4 | 89˚C | 00:01 |
| Step 5 | 87˚C | 00:01 |
| Step 6 | 85˚C | 00:01 |

11. Create T7E1 mix in a 1:9 dilution ratio with dd H2O

| Component | Amount(µl)/Sample |
|---|---|
| T7 Endonuclease I:dd H2O | 2.5 |

12. Add 2.5ul of diluted T7E1 Endonuclease to each tube
13. Vortex then spin down.
14. Incubate tubes at 37°C for 1 hour
15. Create a 1.5% agarose gel
    a. Add 7µl of Loading dye to each sample
    b. Load at least 10µl of product to each well
    c. Add 10µl of ladder to one or more wells
    d. Run the gel at 150V for about 30 minutes

16. Interpretation of gel:

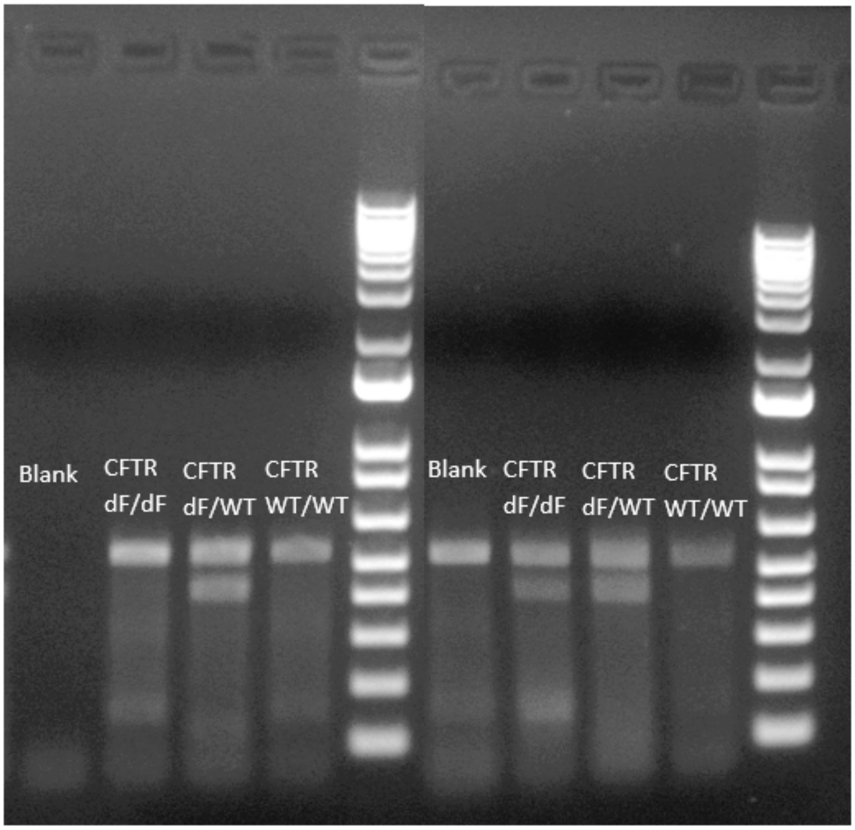


*Figure 1. Control results for CFTR dF without WT DNA addition*

*Figure 2. Control Results for CFTR dF With WT DNA Addition*

a. Gel 1 interpretation
    i. Homozygous: 533 bp
    ii. Heterozygous: 533bp/ 412 bp
    iii. WT: 520 bp
b. Gel 2 Interpretation
    i. Homozygous: 412bp/ 533bp
    ii. Heterozygous: 412bp/ 533bp
    iii. WT: 533 bp

## **Flexivent Data Processing Protocol:**

- Create “Flexivent Script” folder
    - In the “Flexivent Script” folder, create these subfolders:
        - “Current Scripts”
        - “CF Basics Input”
        - “CF Basics Output”
        - “Flow Vol Input”
        - “Flow Vol Output”
    - Within these folders, create a folder for each genotype

How to export the data from flexivent software to a flash drive:

- Open Flexiware software and select database
- Open “Review and Reporting” option
- Select animal and date of experiment
- Select these export scenarios:
    - “CF ferret basics”
    - “Flow Vol”
    - “PV loops”
- Click through all options until it asks for export location
    - Open flashdrive location
    - Create new folder labeled with date of experiment and animal ID

- Create an “Exported Data” folder on your desktop.
- Transfer the files in a folder with date and genotype of ferret. You will have 3 files in the folder.

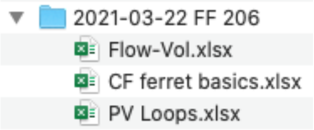


- In a known location in your computer, create these folders for each genotype:

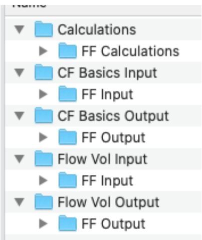


## **Processing of CF Ferret Basics DATA:**

- Open “CF ferret basics” file.
- Delete rows 1-7.
- Delete row 2 on the new file.
- Save it as csv.

- Put it in the “CF Basics Input” folder.
- Open both script windows using ANACONDA Navigator.
    - Go to Jupyter Notebook and press Launch.
- Open both scripts as a new window.
- Go to CF Basics script and replace input and output paths according to the location of the file and file name.
- Run the Script.
- Go to the “CF Basics Output” folder and open the newly created file.
- Make averages of all the measurements. Save as an excel file into the “Calculations” folder.

Flow Vol:
- Open “Flow Vol” data
- Copy rows 8-13 into a new excel file and save as csv
    - Name file with date, animal ID and “header”
- Delete rows 1-14 in original Flow Vol file
- Delete the new row 2
- Save file as date animal ID and Flow Vol
- Put both files into “Flow Vol Inputs” file
- Copy pathway of file into the script
- Run script

Manually calculating flow vol data:
- Identify which NPFE reps failed and mark on original flow vol exported data file
- Use the “Max” function to find the FVC of all valid NPFE (max function of the “Vcw_calc” column)
- Average all reps
- Using the “time” column, find the corresponding times for the FEV:
    - 0.624 (0.4)
    - 0.649 (0.425)
    - 0.674 (0.45)
    - 0.699 (0.475)
- Average all valids reps for each timepoint

Processing of Flow-Vol DATA:

- Open "Flow-Vol" file.
- Copy rows 8-13 and put in a new file.
- Save in the "Flow Vol Input" folder as "date genotype Header.csv".
- Delete rows 1-14 and after that row 2.
- Save in the same folder as "date genotypeID Flow Vol.csv".
- Run the script and go to Flow Vol Output file and open the Summary file.
- Check the Flag column. It should say FALSE! This means there were 6 repetitions. If it says TRUE, it means there were > 6 repetitions. But the script always takes into account the last 6.
- Check FEV0.45 and FVC to see if those numbers are close to the one reported in CF Basics data. There should be at most ~1 ml difference.
- When making summary, use the data reported in Flow Vol Summary file.

Sometimes, when you have an error in any of the loops, you may have to exclude the data manually. To do that, follow this protocol:

- Go to "Flow Vol" file in the exported data folder.
- For each "Vcw_calc" column, calculate MAX.
- Calculate the average of the ones that doesn't have an error. **This represents FVC.**

To find FEV 0.4, FEV 0.425, FEV 0.45, FEV 0.475, add 0.224 to each.

0.4 ➔ 0.624 (row 336)
0.425➔ 0.649 (row 349)
0.45 ➔ 0.674 (row 362)
0.475 ➔ 0.699 (row 375)

- Find the new numbers in the corresponding rows. Highlight them in green.
- Average the "Vcw_calc" for each column. **This represents the hand calculated FEVs.**
- Put this in the project summary master sheet
    - CF Basics results in the calculations folder go to columns H to T.
    - Flow Vol Summary results in the calculations folder go to columns V to AC.

# FlexiVent User Instructions

**<u>Before starting the flexiVent System:</u>**

- Prepare xylazine/ketamine mix.
  - Protocol dose:
    - 0.25-1.5 mg/kg xylazine
    - 5-25 mg/kg ketamine
  - Also bring antisedan (xylazine reversal).
    - Protocol dose: 0.4-1 mg/kg (only IM)
  - Weight ferret and record on anesthesia log. You need this weight at the beginning of the flexivent experiment. If animal lost weight, always use highest weight recorded.
  - Calculate the volume of ketamine/xylazine that can be given and record on the anesthesia log.
- ***<u>Do NOT sedate the animal until flexivent is set up and the intubation tube is calibrated</u>***

**<u>Setting up the experiment:</u>**

Starting the Flexivent: Logon to flexiVent laptop and start the flexiWare 8 software. Turn on the flexiVent, making sure that the ventilator is connected to the laptop via ethernet cable. After the initial start-up screen on the flexiVent, wait until it status says 'idle' before continuing.

- if more than 5 minutes passes without entering an experiment session, the flexiVent automatically turns off. If the flexiVent is not on when the flexiWare begins to communicate with the device, then the software will automatically quit.
- Every $10^{th}$ start up, the flexivent will automatically perform a "self test" to ensure all the valves work correctly.

*Databases:*

The software will start with the main menu showing three different icons to select:

- *Study Definition and Planning*: used for managing test subjects, study parameters, and study groups.
- *Experiment Session*: used to open an experiment database
- *Review and Reporting*: used after an experimentation session to review and export data.

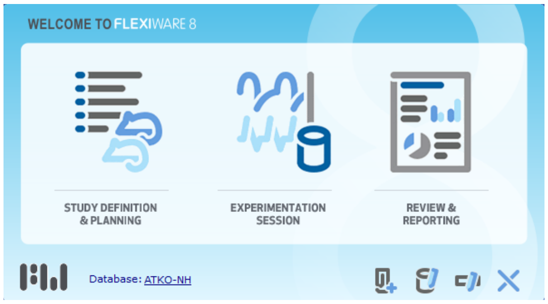


When first opening the Flexivent software, make sure to check that the experimental database is correct. If not, click on the selected database. A screen will prompt the user to choose an experiment database or keep the default. If the default database is correct then click "Ok". If the database is not the correct one, click

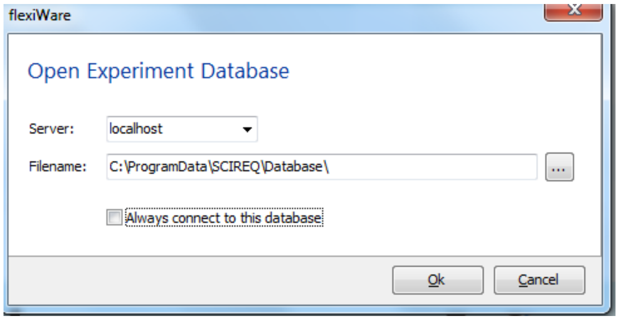


on the underlined database name and select open existing database. Click on the three-dotted button and select the correct database from the list, and the software will once again return to the main menu. To start a new experiment, select *Experimentation Session*.

**<u>Starting the experiment:</u>**

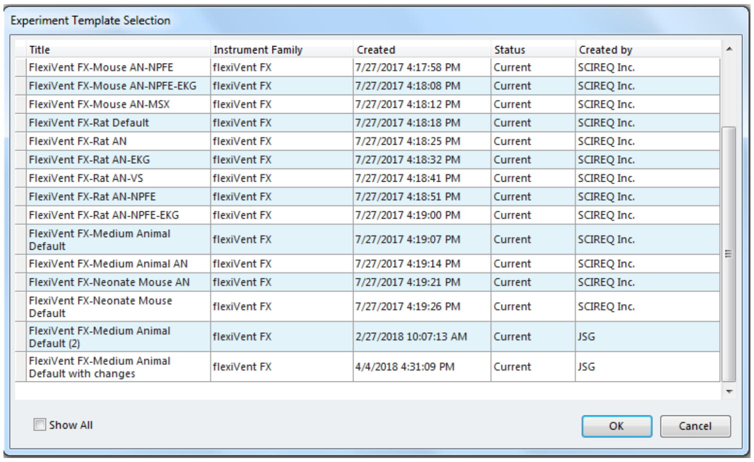


After clicking the *Experimentation Session* tab, the software will prompt to choose template script. If the user alters the template, add initials or name so that the user can be identified by other users. Current template is: Flexivent FX6-Ferret NPFE (2019-05-08) with MW initials. Next, the software will ask for the user to verify that the FX6 module is connected to the device and accept if it is true.

*Subject selection:*

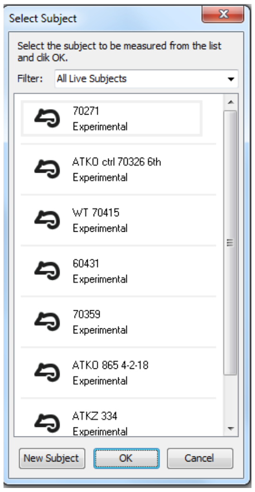


Once the template is selected, the machine will ask for a subject. If the animal has already been tested, find it's ID and hit Ok. It will prompt for the animals current weight next. If the animal currently weighs less than it did the last experimentation session, use the higher weight.

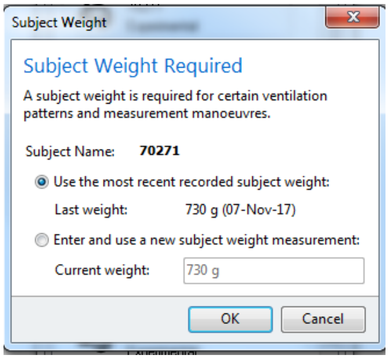


If the animal has not been tested before, click "New Subject" in the lower left corner. Add in the animal's information, including: **<u>FULL</u>** microchip ID, DOB, genotype, gender, and current weight.

***Calibrating the Flexivent:***

The software now moves on to the calibration steps of the channel, plethysmograph chamber, and aeroneb. The channel calibration requires the user to use a manometer add 300 mmMg to the opening that connects the ferret to the flexivent. It will also prompt the user to calibrate without anything attached the connector.

- do not tip manometer or fluid will spill out.

To calibrate the plethysmograph chamber, simply close the chamber when prompted and hit "next". Unless the nebulizer is being used, the aeroneb calibration can be skipped. If the flexivent says the calibration is expired, hit "enter known values" and make some up. It won't affect the experiment as long as the nebulizer isn't used.

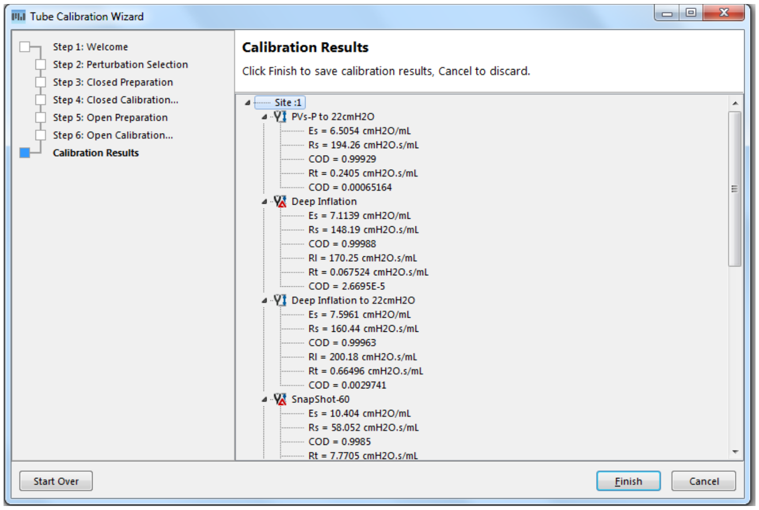


The dynamic tube calibration will prompt the user connect a clean and dry endotracheal tube suited for the selected subject and connect to the Y tubing. Always calibrate the largest tube possible to start. Cover the end hole when prompted, remembering to also cover the second hole if present. Leave tube open when prompted and complete the calibration. If the tube doesn't fit in the animal after trying to intubate, recalibrate with a smaller tube.

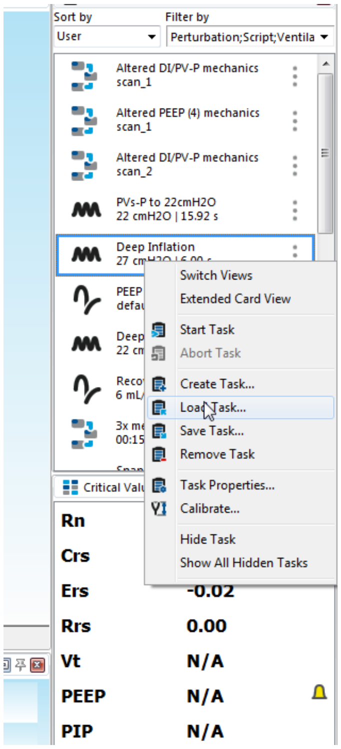


If any of the selected perturbations failed the tube calibration, the user will not be able to perform these perturbations. Alternatively, instead of starting the calibration over when a single perturbation fails, the user can select the perturbation from the right panel to the side in the experiment session view and right click and select calibrate. Follow the prompted steps and check for a passed calibration. If the user needs to calibrate a different endotracheal tube after completing the first calibration, click on the instruments button, then select dynamic tube calibration. This will have the user re-calibrate the endotracheal tube.

There are two types of endotracheal tubes that will work with our ferrets and the flexiVent: white and blue

a. The white endotracheal tube is used on mainly adult animals and inner diameter (I.D.) is 3.0mm and outer diameter (O.D.) is 4.2mm.
b. The blue endotracheal tube is used on mainly really young animals or small females, specs are 2.5mm I.D. and 4.0mm O.D.

If intubation seems difficult with the largest endotracheal tube size, recalibrate with next smaller size and try intubation again. NOTE: the cuff must be inflated in order for the lung to become pressurized and the user to get any real data from the flexiVent.

Channel calibrations need to be performed every 14 days or every time the flexiVent is moved from one location to another. The aeroneb attachment is needs to be calibrated (characterized) every 42 days. Dynamic tube calibration needs to be done for each animal, for each session, with the specific endotracheal tube going to be used for intubation.

If the nebulizer is not being used, this part can be skipped and values can just be randomly added by clicking "Enter known values". Aeroneb characterization requires a drying tube filled with Didrite and the aeroneb nebulizer connected to the flexiVent. A

volume of saline is inserted into the nebulizer and both the drying tube and the nebulizer containing saline are pre-weighed. Scale needs to be to the 1000th of a gram. Follow the directions for characterization and re-weigh both drying tube and nebulizer when finished. If results are accurate (may take 1-5 tries), then the user may proceed with the nebulizer experiment.

*Ventilation:*
After the dynamic tube calibration is performed, the software will ask the user if they would like to start default ventilation pattern, either yes or no can be selected. If the user clicks no, the ventilation can be started by clicking “Medium animal default”. Keep the isoflurane at 5% until the animal is breathing passively. This is shown by the blue waveform at the bottom of the screen. If it does not align with the red line above, the animal is not passive yet. Once that does occur, decrease the isoflurane to 2%.

The level of isoflurane should not be below 2% while conducting any experiment on the flexiVent. Always check that the flow gauge from oxygen concentrator is at 3L/min and from the isoflurane vaporizer is 1L/min. If the animal is young (<6months) or if particularly resistant to anesthesia, the level of isoflurane can be increased to 5% until the animal is completely passive.

- *that animals with obstructive lung disease (emphysema or CF) may exhibit delayed or erratic isoflurane clearance. This can mean that the ferret will either be slower to wake up or can even become sedated again after awakening. Be careful as you adjust the isoflurane, try to use the least amount above 2% minimum for the ferret. If you must increase to a high level to quickly sedate, decrease it after passive to a level just above what it was before.*
  Example: Your ferret is not passive at 2% and needs 4% isoflurane to get sedated. Once it is calm and passive, immediately drop isoflurane to 2.5-3% and observe for a time to ensure it stays passive. This will be less anesthetic that has to wear off later.

*Script selection:*
Select the script based on the size of the animal. Females and males under 4 months of age will use the “small ferret script”. Large males have an inspiratory capacity that can be greater than the flexiVent’s max capacity of 82-83mL. For these animals, the user will need to run the “Large Ferret Script” which gives a multi-step approach to calculating inspiratory capacity. When data is exported, the last two numbers of each Ferret IC SS vs MS script values can be added together for the larger, total combined inspiratory capacity measurement. **Make sure to log vitals, including temperature, before starting the script.**

**<u>During data collection:</u>**

Monitor vitals (HR, respiration, isoflurane, and note time) every 10-12 minutes. If HR cannot be obtained, note the color of the mouth mucosa (should be pink). Log all vitals on anesthesia log. In lab notebook, note animal ID, length, weight, tube size, and select flexivent parameters. If a pretubation fails, note which perturbation and which rep in the lab notebook.

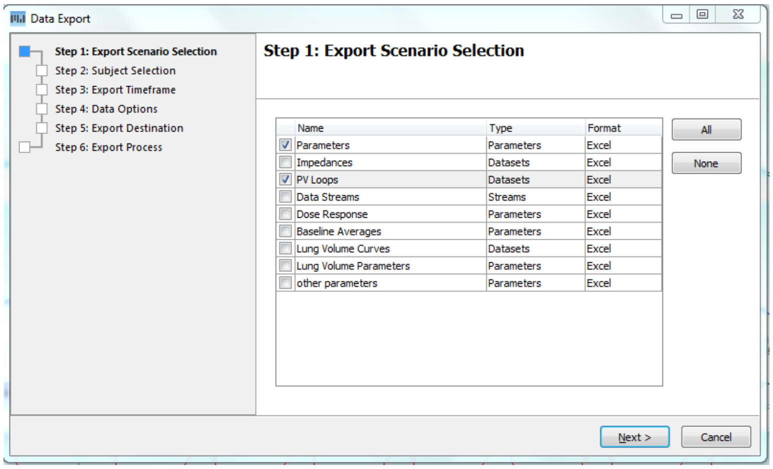


## Exporting data:

To export the data from the current session, go to *File→export data*. The software will pop-up with a window that walks the user through different export preferences for the current data set.

c. Step 1: Export Scenario Selection—Select which items are to be exported. Normal experiments, only choose *CF ferret basics, Flow Vol* and *PV Loops*.

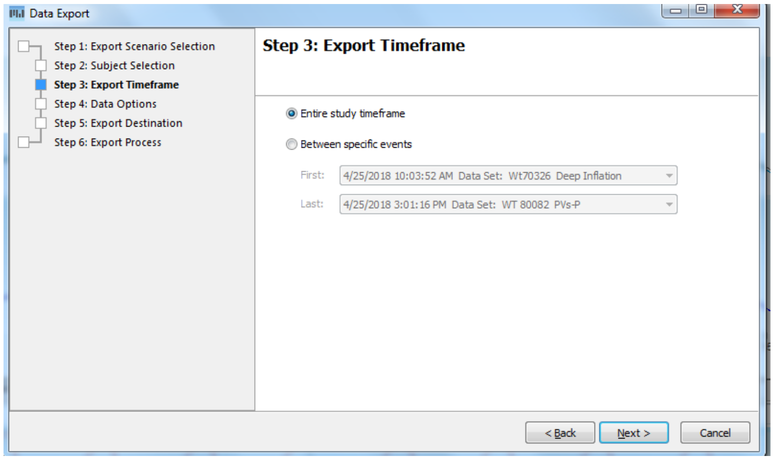


d. Step 2: Subject Selection—just click next, the software will only allow user to export data that was collected during the current experiment session.

e. Step 3: Export Timeframe—can export entire study timeframe; this will include all animals that were run during the current experimental session and combine into a single excel file. If the user prefers to have each animal as a separate file, then select *Between specific events* and select when the perturbations start for the animal and end for that animal.

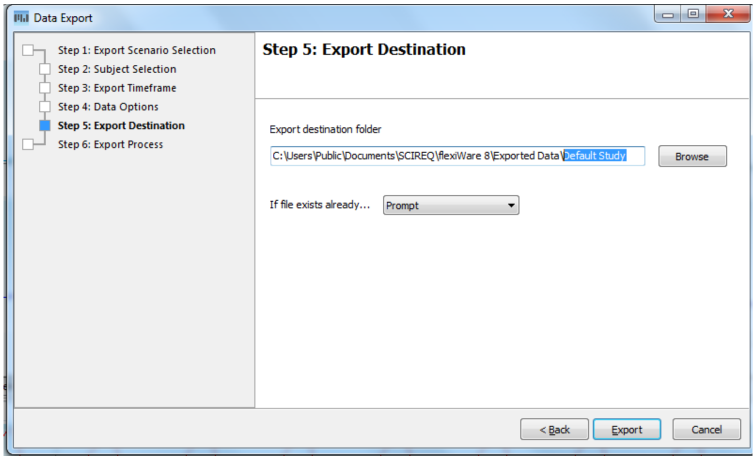


f. Step 5: Export Destination—the software will export data into separate files for each export scenario into a folder under the current study's title. To export to a specific folder, select

"Browse". Then make a new folder labeled with the study date and animals included in that export

g. Step 6: Export process—Each export scenario will appear in the folder as a Microsoft excel file, with a generic name like *Parameters*.

To export data later, open the Flexiware software, select the database with the correct animal, and select the *Review and Reporting* option. From there, you can select the specific study date and export as if it was directly being exported from a session.

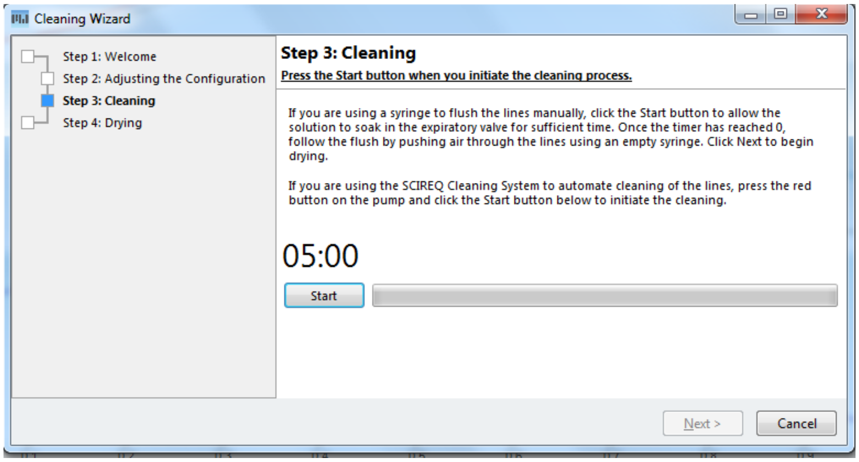


**Cleaning the flexiVent:**

After the experimental session is complete and the data has been exported, the user can now conclude the experiment with cleaning of the flexiVent. This only needs to be performed once per day, after each session is complete. Click to exit the program (upper right) and the software will prompt the cleaning wizard. Supplies needed: 3 beakers (one at least 2 L) 2 sets of connecting tubing, and one 30mL syringe; one of the smaller ones filled half-way with water and the other smaller one filled half-way with soapy water. Connect first set of tubing to right side of expiratory port (front of flexiVent) and the other to the bottom of the two side ports (also expiratory). NOTE: DO NOT TRY AND PLACE ANY TUBES IN THE INSPIRATORY PORTS AS THIS CAN RUIN THE FLEXIVENT. Fill syringe first with soap water and push through first tube going through flexiVent to side expiratory port into the receiving beaker. Switch to clean water after all the soapy water is gone. When the 5 mins are up, put the FX adapter with closed circuit Y tubing in place and start drying cycle. When the 10 mins are up, the software can be closed and the flexiVent shut off and stored.

**Data Analysis:** After the data has been exported, it will show up as an excel spreadsheet, organized chronologically with specific event ID's and further organized by parameter.

**Note for transport of flexiVent:**

The flexiVent needs to have the piston locked into place before transport and unlocked upon arrival before start of the experiment. The screw will need to be completely removed in order to lock the piston into place.

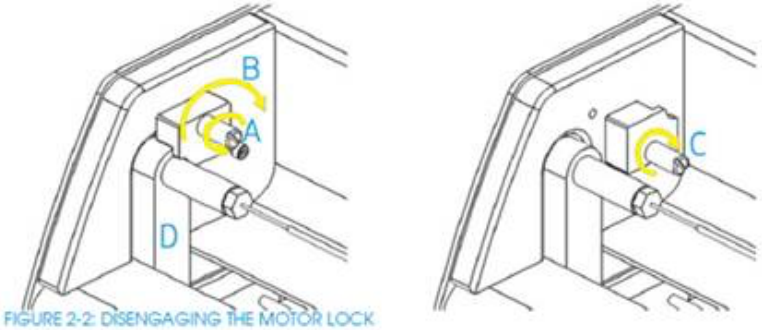


FIGURE 2-2: DISENGAGING THE MOTOR LOCK

**Sharing of databases:** Databases are large and need to be shared via another service than email (the server or pcloud…). Databases can be accessed through the following path: C:\ProgramData\SCIREQ\Database.

**Editing/configuring the system:** Open the flexiWare software and click on edit the system icon (rectangle with pencil shape). To connect/recognize the specific flexiVent, go to instrument properties and connect. To configure the system, go to system properties and configure instrument to device.
